# Open-air green-light-driven ATRP enabled by dual photoredox/copper catalysis†

**DOI:** 10.1039/d2sc04210j

**Published:** 2022-09-20

**Authors:** Grzegorz Szczepaniak, Jaepil Jeong, Kriti Kapil, Sajjad Dadashi-Silab, Saigopalakrishna S. Yerneni, Paulina Ratajczyk, Sushil Lathwal, Dirk J. Schild, Subha R. Das, Krzysztof Matyjaszewski

**Affiliations:** Department of Chemistry, Carnegie Mellon University Pittsburgh Pennsylvania 15213 USA gszczepa@andrew.cmu.edu matyjaszewski@cmu.edu; Faculty of Chemistry, University of Warsaw Pasteura 1 02-093 Warsaw Poland; Department of Biomedical Engineering, Carnegie Mellon University Pittsburgh Pennsylvania 15213 USA; Faculty of Chemistry, Adam Mickiewicz University, Uniwersytetu Poznańskiego 8 61-614 Poznań Poland; Center for Nucleic Acids Science & Technology, Carnegie Mellon University Pittsburgh Pennsylvania 15213 USA

## Abstract

Photoinduced atom transfer radical polymerization (photo-ATRP) has risen to the forefront of modern polymer chemistry as a powerful tool giving access to well-defined materials with complex architecture. However, most photo-ATRP systems can only generate radicals under biocidal UV light and are oxygen-sensitive, hindering their practical use in the synthesis of polymer biohybrids. Herein, inspired by the photoinduced electron transfer-reversible addition–fragmentation chain transfer (PET-RAFT) polymerization, we demonstrate a dual photoredox/copper catalysis that allows open-air ATRP under green light irradiation. Eosin Y was used as an organic photoredox catalyst (PC) in combination with a copper complex (X–Cu^II^/L). The role of PC was to trigger and drive the polymerization, while X–Cu^II^/L acted as a deactivator, providing a well-controlled polymerization. The excited PC was oxidatively quenched by X–Cu^II^/L, generating Cu^I^/L activator and PC˙^+^. The ATRP ligand (L) used in excess then reduced the PC˙^+^, closing the photocatalytic cycle. The continuous reduction of X–Cu^II^/L back to Cu^I^/L by excited PC provided high oxygen tolerance. As a result, a well-controlled and rapid ATRP could proceed even in an open vessel despite continuous oxygen diffusion. This method allowed the synthesis of polymers with narrow molecular weight distributions and controlled molecular weights using Cu catalyst and PC at ppm levels in both aqueous and organic media. A detailed comparison of photo-ATRP with PET-RAFT polymerization revealed the superiority of dual photoredox/copper catalysis under biologically relevant conditions. The kinetic studies and fluorescence measurements indicated that in the absence of the X–Cu^II^/L complex, green light irradiation caused faster photobleaching of eosin Y, leading to inhibition of PET-RAFT polymerization. Importantly, PET-RAFT polymerizations showed significantly higher dispersity values (1.14 ≤ *Đ* ≤ 4.01) in contrast to photo-ATRP (1.15 ≤ *Đ* ≤ 1.22) under identical conditions.

## Abstract 2

Fully oxygen-tolerant photoinduced atom transfer radical polymerization (photo-ATRP) allowed the synthesis of well-defined polymers using a Cu catalyst and eosin Y at ppm levels in both aqueous and organic media.

## Graphical abstract

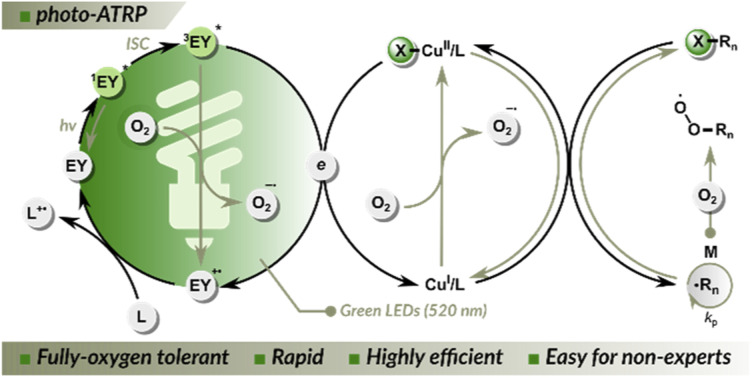

## Introduction

Reversible-deactivation radical polymerization (RDRP) has been recognized by IUPAC as one of the most important emerging technologies in chemistry that could change our world.^1^ The key RDRP techniques are reversible addition fragmentation chain-transfer (RAFT) polymerization and atom transfer radical polymerization (ATRP).^2–4^ Both of these methods allow the polymerization of various vinyl monomers under mild conditions, giving unprecedented control over polymer architecture.^5–7^

**Fig. 1:**
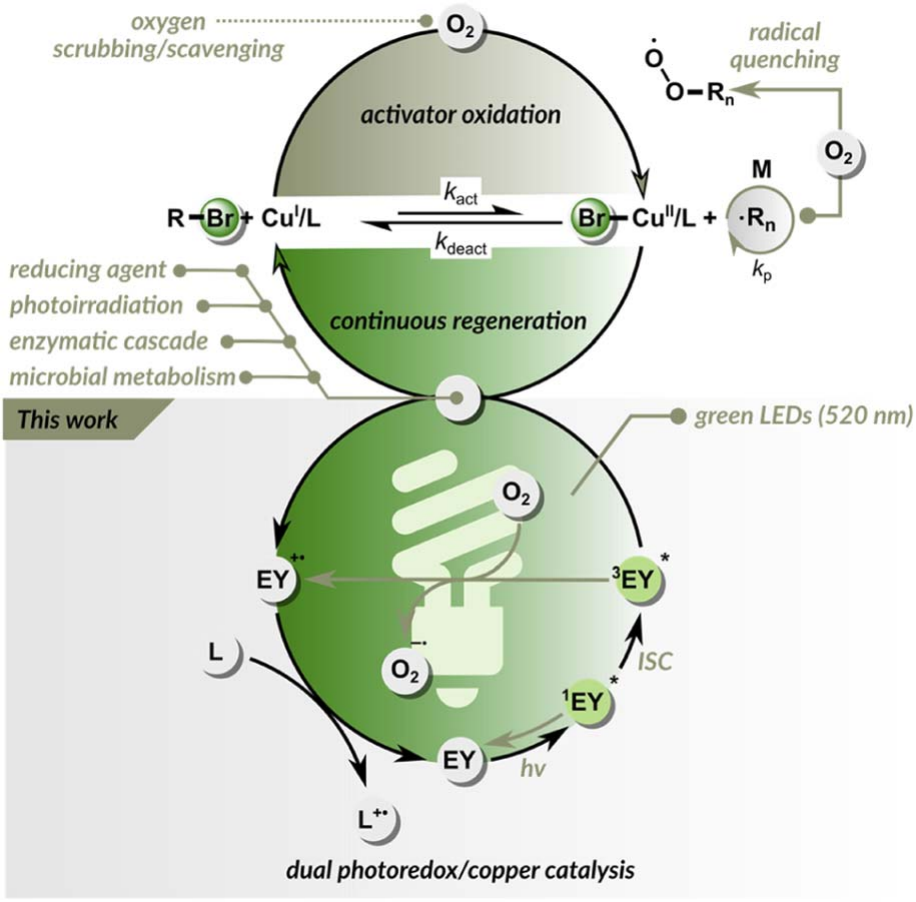
Approaches for attaining oxygen tolerance in ATRP.

ATRP is a reversible redox process, typically catalyzed by copper complexes.^8,9^ The control over radical propagation in Cu-catalyzed ATRP is provided *via* a reversible redox reaction (Fig. 1). First, the Cu^I^/L catalyst (L is typically a polydentate amine ligand) reacts with the dormant C(sp^3^)–X polymer chain end through a concerted inner sphere electron transfer to form two species: a X–Cu^II^/L deactivator and a propagating carbon-based radical. Then, in the reverse reaction, the propagating radical reacts with the X–Cu^II^/L, recovering the active form of the catalyst (Cu^I^/L) and the dormant chain end (C(sp^3^)–X).^10–12^ A limitation of ATRP, like any other RDRP technique is its sensitivity to oxygen, as both initiating and propagating radicals are quenched by oxygen. Furthermore, molecular oxygen can oxidize an ATRP catalyst to its inactive form, inhibiting polymerization. Therefore, normal ATRP requires strictly anaerobic conditions.^13^ The sensitivity of ATRP techniques to oxygen hinders their use under ambient conditions and necessitates deoxygenation by inert gas purging, freeze–pump–thaw cycles, or using a glovebox. As a result, ATRP methods are time-consuming and can be challenging for non-experts.

To address these challenges, many efforts have been made to increase oxygen tolerance in ATRP.^14–16^ Since, at the ATRP equilibrium, the concentration of Cu^I^/L is much higher than the concentration of propagating radicals, oxygen predominantly oxidizes Cu^I^/L rather than reacting with radicals. This inspired researchers to harness Cu^I^/Cu^II^ catalysis to function also as an oxygen scavenger.^17–22^ The continuous reduction of Cu^II^/L back to Cu^I^/L makes the ATRP inherently resistant to oxygen (Fig. 1).^14^ The alternative approach is based on adding glucose oxidase enzyme that removes oxygen without affecting the polymerization process, as in enzyme-assisted ATRP techniques.^23–26^ Despite these great improvements, most modern ATRP techniques tolerate only a limited amount of oxygen and can be performed in sealed vessels.^22,27–31^ When an open reaction vessel is used, the regeneration of the catalyst is usually slower than oxygen diffusion into the reaction site. To date, only a few ATRP systems are fully oxygen-tolerant and can be run in open-air reaction vessels.^32–37^

Photoinduced ATRP (photo-ATRP) techniques use light energy to generate activator Cu^I^/L species, thus initiating polymerization.^38–44^ They typically require the use of biocidal UV light (<400 nm), which can degrade proteins, damage DNA, or initiate unwanted side reactions.^45^ Organocatalyzed ATRP (O-ATRP) relies on direct activation of the dormant polymer chain end by electron transfer from the photocatalyst in an excited state.^46–52^ It allows polymerization over a broad visible light spectrum,^53–56^ but moderate control and limited scope of monomers hinder its practical application, particularly under biologically relevant conditions.^57^ Dual catalytic ATRP systems use ppm-level copper catalysts to attain a controlled polymerization process in the ground state and photoredox catalysts (PCs) to reduce deactivators *via* photoinduced electron transfer, maintaining radical propagation.^58–65^ Moreover, when a PC in the excited state has sufficient redox potential, it can react directly with a dormant C(sp^3^)–X polymer chain end, generating radicals and thus offering an additional activation pathway. These methods are highly efficient under long-wavelength light, but have limited oxygen tolerance.

Another powerful photochemically driven RDRP technique is the photoinduced electron transfer RAFT (PET-RAFT) polymerization method developed by Boyer and co-workers.^66^ In the PET-RAFT, an excited PC can transfer energy/electrons to a chain transfer agent (CTA), generating propagating radicals.^67–69^ As in the conventional RAFT process, control over polymerization is provided by a CTA *via* a degenerative chain transfer mechanism.^3^ PET-RAFT can be used to polymerize a wide range of monomers, is oxygen tolerant, and exhibits perfect temporal control. Initially, PET-RAFT was performed using tris(phenylpyridinium) iridium(iii) catalyst.^66^ The method was later expanded to several other photoredox systems, allowing polymerization under longer wavelength light.^70–76^ Eosin Y is one of the most widely used photocatalysts,^77–79^ especially for biological applications, due to its solubility in water, low toxicity, and cost. PET-RAFT catalyzed by eosin Y has recently been used to engineer protein and cell surfaces.^45,80^

Here, we demonstrate a dual photoredox/copper catalysis that enables ATRP under green light irradiation (Fig. 1). Inspired by PET-RAFT polymerization, we used eosin Y as the PC. In this dual catalytic system, control over radical propagation is provided by ATRP equilibrium, while eosin Y is essential for triggering and maintaining polymerization. In addition, the dual photoredox/copper catalysis removes oxygen, enabling the rapid open-to-air synthesis of well-defined polymers in both aqueous and organic media, outperforming PET-RAFT under identical conditions.

## Results and discussion

### EY/Cu dual catalysis: optimization of ATRP conditions

**Fig. 2:**
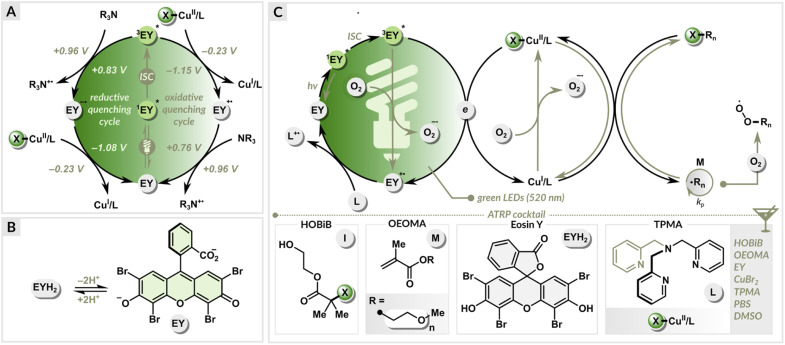
(A) Reductive quenching *vs.* oxidative quenching cycle, (B) formation of EY in PBS solution, (C) proposed mechanism.

A set of polymerizations was performed to evaluate the influence of the dual photoredox/copper catalysis on the ATRP process. Oligo(ethylene glycol) methyl ether methacrylate (average *M*_n_ = 500, OEOMA_500_) monomer was polymerized under green LEDs (520 nm, 9.0 mW cm^−2^), using 2-hydroxyethyl 2-bromoisobutyrate (HOBiB) as the initiator, eosin Y in neutral form (EYH_2_) as the organic photoredox catalyst and CuBr_2_/TPMA (TPMA = tris(2-pyridylmethyl)amine) as the precatalyst (Fig. 2). TPMA was used as a ligand since it forms a stable Cu^I^/TPMA complex in water, enabling a well-controlled polymerization of methacrylates.^81^ The polymerizations were conducted in phosphate-buffered saline (PBS) with DMSO (10% v/v) in open vials (Fig. S1†).

Photo-ATRP was first attempted using molar ratios of [OEOMA_500_]/[HOBiB]/[CuBr_2_]/[TPMA] = 200/1/0.2/0.6. As expected, no OEOMA_500_ conversion, as measured by ^1^H NMR, was observed in the absence of the photoredox catalyst (entry 1, Table 1). O-ATRP catalyzed with the EY/TPMA (without CuBr_2_) enabled rapid polymerization, reaching 89% monomer conversion within 30 min (entry 2, Table 1),^56^ but size exclusion chromatography (SEC) analysis showed that the polymer had a high dispersity (*Đ*) of 4.30. On the other hand, when EYH_2_ was used in combination with CuBr_2_ and excess TPMA ligand, high monomer conversion (88%) and well-controlled polymerization (*Đ* = 1.19) were achieved (entry 3, Table 1). These experiments confirmed the critical role of dual catalysis in ensuring a well-controlled polymerization under green light irradiation.

**Table 1:** EY/Cu dual catalysis: optimization of ATRP conditions^a^

No.	EYH_2_ (equiv)	CuBr_2_ (equiv)	TPMA (equiv)	rpm^b^	Conv.^c^ (%)	*M*_n,th_	*M*_n,abs_ d	*M*_n,app_ e	*Đ* e
1	—	0.2	0.6	0	0	—	—	—	—
2	0.05	—	0.6	0	89	89 000	187 500	126 000	4.30
3	0.05	0.2	0.6	0	88	88 000	84 000	65 000	1.19
4	0.05	0.2	0.6	250	86	86 000	82 500	64 000	1.18
5	0.05	0.2	0.6	500	86	86 000	86 000	66 000	1.15
6	0.05	0.2	0.6	1000	86	86 000	86 000	66 000	1.18
7	0.1	0.2	0.6	500	89	89 000	89 500	68 500	1.20
8	0.01	0.2	0.6	500	84	84 000	79 500	62 000	1.15
9	0.005	0.2	0.6	500	80	80 000	73 000	58 000	1.16
10	0.01	0.3	0.6	500	74	74 000	70 000	56 000	1.14
11	0.01	0.1	0.6	500	92	92 000	90 500	69 000	1.21
12	0.01	0.2	0.4	500	77	77 000	61 000	50 000	1.19
13	0.01	0.2	1.2	500	91	91 000	90 500	69 000	1.16

a Reactions conditions: [OEOMA_500_]/[HOBiB]/[EYH_2_]/[CuBr_2_]/[TPMA] = 200/1/x/x/x, [OEOMA_500_] = 300 mM, in PBS with DMSO (10% v/v), irradiated for 30 min under green LEDs (520 nm, 9.0 mW cm^−2^) in an open vial with stirring at 0–1000 rpm.

b Revolutions per minute (rpm).

c Monomer conversion was determined by using ^1^H NMR spectroscopy.

d Molecular weight (*M*_n,abs_) was determined by Mark–Houwink calibration.

e Apparent molecular weight (*M*_n,app_) and dispersity (*Đ*) were determined by GPC analysis (DMF as eluent) calibrated to poly(methyl methacrylate) standards.

To evaluate oxygen tolerance, the stirring rate was increased from 0 to 250, 500, and 1000 rpm (entries 4–6, Table 1). All polymerizations were successful, yielding well-defined polymers with low dispersity (*Đ* < 1.18) and controlled molecular weights, indicating that the EY/Cu system is highly tolerant to increased oxygen mass transfer.

The amount of EYH_2_ was then varied to explore the performance of the dual catalytic system (entries 7–9, Table 1). Increasing the EYH_2_ concentration from 75 μM to 150 μM resulted in 89% monomer conversion and slightly higher dispersity (*Đ* = 1.20; entry 7, Table 1). In contrast, a 5-fold decrease in EYH_2_ concentration to 15 μM caused only a slight decrease in conversion (84%) while improving control over the polymerization (*M*_n,th_ = 84 000, *M*_n,abs_ = 79 500, *Đ* = 1.15; entry 8, Table 1). After further reduction of EYH_2_ to 7.5 μM (25 ppm relative to the monomer), the dual EY/Cu catalysis still provided a well-controlled polymerization (*Đ* = 1.16) with predetermined molecular weight (*M*_n,th_ = 80 000, *M*_n,abs_ = 73 000) and high monomer conversion of 80% (entry 9, Table 1).

Finally, the effect of CuBr_2_ and TPMA ligand concentrations was investigated (entries 10–13, Table 1). Increasing the amount of copper diminished the polymerization rate while improving its control (conv. = 74%, *Đ* = 1.14; entry 10, Table 1). Increasing the ligand concentration led to higher monomer conversion (conv. = 91%) and dispersity of 1.16 (entry 13, Table 1).

### Proposed mechanism

The neutral form of eosin Y with spirocyclic structure (EYH_2_) exhibits low absorbance in the visible region.^82,83^ In PBS solution, EYH_2_ undergoes sequential deprotonation leading to the photoactive ring-opened form (EY) (Fig. 2), which exhibits an absorption maximum at ∼520 nm. Under green light irradiation, EY in the excited triplet state (^3^EY*) can both accept (*E*_1/2_(^3^EY*/EY˙^−^) = +0.83 V *vs.* SCE) or donate an electron (*E*_1/2_(EY˙^+^/^3^EY*) = −1.15 V *vs.* SCE) (Fig. 2A).^83^ In the reductive quenching cycle, ^3^EY* is quenched by accepting an electron from an ATRP ligand (with a tertiary nitrogen atom), which acts as a sacrificial electron donor. This results in the formation of the EY radical anion (EY˙^−^) and an amine radical cation (L˙^+^). The formed EY˙^−^ (*E*_1/2_(EY/EY˙^−^) = −1.08 V *vs.* SCE) then donates an electron to X–Cu^II^/L, generating Cu^I^/L activator and EY in the ground state, completing the photocatalytic cycle. In the oxidative quenching cycle, ^3^EY* is quenched by donating an electron to Cu^II^/L, leading to the formation of EY˙^+^ and Cu^I^/L. Finally, the photocatalytic cycle is closed by reducing the oxidized EY (*E*_1/2_(EY˙^+^/EY) = +0.76 V *vs.* SCE) with an alkylamine.

The free energy of a photoinduced electron transfer can be determined using the Gibbs energy of photoinduced electron transfer equation:Δ*G*_et_ (eV) = −[*E*_1/2_(*A*/*A*˙^−^) − *E*_1/2_(*D*˙^+^/*D*)] − *E*_PC*_ + Δ*E*where *E*_1/2_(*A*/*A*˙^−^) is the reduction potential of an electron acceptor (*A*), *E*_1/2_(*D*˙^+^/*D*) is the oxidation potential of a sacrificial electron donor (*D*), *E*_PC*_ is the energy of the singlet or triplet excited state of a photocatalyst, Δ*E* = <0.1 eV, and is often neglected in photophysical estimations. For EY, the excitation energy of ^3^EY* is 1.91 eV. Thus, for the oxidative quenching:Δ*G*_et_ (eV) = −[*E*_1/2_(Cu^II^L/Cu^I^L) − *E*_1/2_(EY˙^+^/EY)] − *E*_EY*_where *E*_1/2_(Cu^II^L/Cu^I^L) = −0.23 V *vs.* SCE, electron transfer from ^3^EY* to Br–Cu^II^/TPMA gives Δ*G*_et_ = −0.92 eV (−21.2 kcal mol^−1^). In contrast, for the reductive quenching:Δ*G*_et_ (eV) = −[*E*_1/2_(EY/EY˙^−^) − *E*_1/2_(*L*˙^+^/*L*)] − *E*_EY*_assuming that the redox potential of TPMA ligand is close to Et_3_N (*E*_1/2_(Et_3_N˙^+^/Et_3_N) = +0.96 V *vs.* SCE), Δ*G*_et_ can be estimated at +0.13 eV (+3.0 kcal mol^−1^). These thermodynamic calculations indicate that the oxidative quenching of ^3^EY* is more favorable than reductive quenching (Fig. 2C). Furthermore, fluorescence quenching experiments showed that the excited state of EY was strongly quenched upon adding Br–Cu^II^/TPMA (Fig. S3A†), while only a slight decrease in fluorescence was observed in the presence of TPMA ligand (Fig. S3B†). Recent mechanistic studies on photoinduced CuAAC (Cu-catalyzed azide–alkyne cycloaddition) reaction triggered by EY also strongly support the oxidative quenching cycle.^84^

In the EY/Cu dual catalysis, both Cu^I^/L and ^3^EY* can react with molecular oxygen (*E*_1/2_(O_2_/O_2_˙^−^) = −0.87 V *vs.* SCE). The continuous regeneration of the Cu^I^/L activator by EY provides high oxygen tolerance (Fig. 2C). The control over radical propagation is achieved through a reversible redox equilibrium between Cu(i) and Cu(ii) complexes, where they act as intermittent activators of dormant species and deactivators of radicals. EY and the ligand used in excess are essential to initiate and sustain green light-induced polymerization in the presence of oxygen.

### Comparison of EY/Cu-catalyzed ATRP with PET-RAFT

Since EY/Cu-catalyzed ATRP is based on the same photoredox catalysis as PET-RAFT polymerization triggered by EY, we were interested in directly comparing the two methods. Such comparisons are rare, and to our knowledge have been reported only for conventional RAFT.^2,85,86^ Moreover, PET-RAFT polymerization is considered one of the most efficient biocompatible oxygen-tolerant polymerization techniques.^4,45^ Thus, a detailed comparison of the two methods is warranted.

**Fig. 3:**
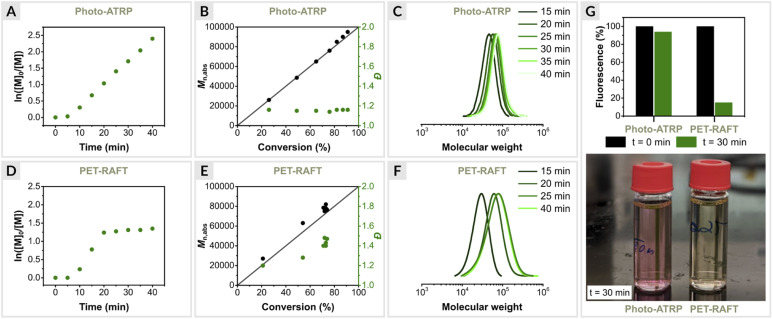
Polymerization kinetics of OEOMA_500_, (A–C) EY/Cu mediated photo-ATRP and (D–F) EY-catalyzed PET-RAFT polymerization. (G) EY fluorescence intensity measurements.

First, photo-ATRP was performed under optimized conditions ([OEOMA_500_]/[HOBiB]/[EYH_2_]/[CuBr_2_]/[TPMA] = 200/1/0.01/0.2/0.6) at a stirring rate of 500 rpm in an open vial. Kinetic analysis revealed a linear relationship between ln([*M*]_0_/[*M*]_*t*_) and time with a short induction period of 5 min, followed by a rapid polymerization that reached 91% monomer conversion within 40 min (Fig. 3A). In addition, the molecular weights increased as a function of monomer conversion, and molecular weight distribution values remained low (*Đ* ≤ 1.16) during the reaction (Fig. 3B and C).

Next, PET-RAFT polymerization of OEOMA_500_ was performed under identical conditions using 4-cyano-4-(phenylcarbonothioylthio)pentanoic acid (CPADB) as the CTA, EY as the photoredox catalyst and TEOA (TEOA = triethanolamine) as the sacrificial electron donor with [OEOMA_500_]/[CPADB]/[EYH_2_]/[TEOA] molar ratios of 200/1/0.01/0.6. Similar to photo-ATRP, a short induction period of 5 min was observed, followed by rapid polymerization, which reached 71% monomer conversion within 20 min (Fig. 3D). However, no further increase in conversion was observed over time. Fluorescence intensity measurements showed an 85% decrease in EY fluorescence after 30 min of PET-RAFT polymerization, while only 6% of the initial amount of EY was photobleached in photo-ATRP (Fig. 3G). The better performance of the EY/Cu dual catalysis can be attributed to the rapid electron transfer from ^3^EY* to Cu^II^/L complex, increasing the long-term stability of EY under green light irradiation. In the absence of a Cu^II^/L, EY degrades faster, leading to the inhibition of PET-RAFT polymerization. In addition, PET-RAFT polymerizations generated polymers with significantly higher dispersity values (*Đ* ≤ 1.45) (Fig. 3E and F), in contrast to photo-ATRP (*Đ* ≤ 1.16).

### Varying targeted degrees of photo-ATRP and PET RAFT polymerization

**Fig. 4:**
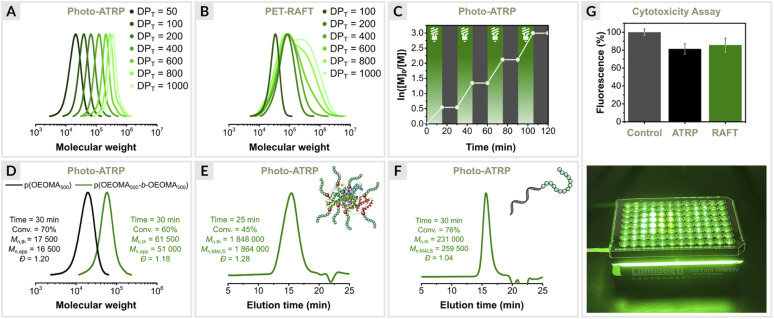
Varying targeted degrees of polymerization, (A) EY/Cu mediated photo-ATRP, (B) EY-catalyzed PET-RAFT polymerization. (C) Temporal control. (D) Chain extension. Synthesis of biohybrids, (E) BSA-p(OEOMA_500_), (F) DNA-p(OEOMA_500_). (G) Comparison of the cytocompatibility of photo-ATRP with PET-RAFT polymerization.

The EY/Cu system was further evaluated for the synthesis of polymers with different molecular weights (Table 2). The target degrees of polymerization (DP_T_) were varied by adjusting the initiator concentration, while the concentrations of the other polymerization components were fixed for each reaction. The results showed a high degree of control for a wide targeted DP range (50–1000) (entries 1–7, Table 2 and Fig. 4A). The monomer conversions reached 69–84% within 30 min, and dispersities remained very low (1.15 ≤ *Đ* ≤ 1.22). However, deviations from the theoretical molecular weights were observed for DP_T_ > 600 (entries 6 and 7, Table 2), which can be attributed to the presence of oxygen. For DP_T_ = 800 and 1000, the initiator concentration (∼0.3 mM) was close to the oxygen concentration in the reaction mixture (∼0.2 mM). The kinetic experiments (Fig. 3A) and oxygen concentration measurements (see later section) suggest that the initiator reacts with oxygen in the initial phase of the reaction when the oxygen concentration in the reaction mixture is highest. This explains the increase in the molecular weight of the polymers obtained (entries 4–7, Table 2). Nevertheless, the polymerizations proceeded with excellent control (*Đ* ≤ 1.16), indicating that the loss of the initiator occurs mainly during the inhibition period, while during polymerization, oxygen is removed by EY/Cu catalysis.

**Table 2:** EY/Cu mediated photo-ATRP of OEOMA_500_ with varying degrees of polymerization^a^

No.	[OEOMA_500_]/[HOBiB]/[EYH_2_]/[CuBr_2_]/[TPMA]	[HOBiB] (mM)	Conv.^b^ (%)	*M*_n,th_	*M*_n,abs_ c	*M*_n,app_ d	*Đ* d
1	50/1/0.0025/0.05/0.15	6.0	74	18 500	16 500	17 000	1.22
2	100/1/0.005/0.1/0.3	3.0	81	40 500	38 000	34 000	1.16
3	200/1/0.01/0.2/0.6	1.5	84	84 000	79 500	62 000	1.15
4	400/1/0.02/0.4/1.2	0.75	79	158 000	179 000	121 000	1.16
5	600/1/0.03/0.6/1.8	0.5	73	219 000	305 000	188 000	1.16
6	800/1/0.04/0.8/2.4	0.325	68	272 000	483 000	275 000	1.15
7	1000/1/0.05/1.0/3.0	0.3	69	345 000	607 000	332 000	1.15

a Reaction conditions [OEOMA_500_] = 300 mM, [HOBiB] = 6.0–0.3 mM, [EYH_2_] = 15 μM, [CuBr_2_] = 0.3 mM, [TPMA] = 0.9 mM in PBS with DMSO (10% v/v), irradiated for 30 min under green LEDs (520 nm, 9.0 mW cm^−2^) in an open vial with stirring at 500 rpm.

b Monomer conversion was determined by using ^1^H NMR spectroscopy.

c Molecular weight (*M*_n,abs_) was determined by Mark–Houwink calibration (see ESI).

d Apparent molecular weight (*M*_n,app_) and dispersity (*Đ*) were determined by GPC analysis (DMF as eluent) calibrated to poly(methyl methacrylate) standards.

PET-RAFT polymerizations were then performed under identical conditions (Table 3). The target DP was set by adjusting the CTA concentration, while the concentrations of the other reagents were fixed for all experiments. The results showed an increase in molecular weight distribution values with increasing DP_T_ (entries 1–7, Table 3 and Fig. 4B). In addition, significant deviations from the theoretical molecular weights were observed. Only for DP_T_ = 100, the polymerization was well controlled (*M*_n,abs_ = 32 000, *Đ* = 1.14). The loss of control over polymerization could be attributed to a very low CTA concentration. Similar to photo-ATRP, a CTA can react with oxygen in the initial phase of the reaction, resulting in its degradation. In the case of RAFT, a CTA not only determines the initial target polymer chain length but is also responsible for the degenerative chain transfer process. In ATRP, control over polymerization is provided by a Cu^II^/L deactivator, which is resistant to oxygen. In addition, Cu catalysis increases the tolerance of ATRP to oxygen since Cu^I^/L species can scavenge oxygen. Therefore, in ATRP, deviations from theoretical molecular weights are observed for higher targeted DT_T_ (entries 5–7, Table 2). At the same time, control over polymerization is not affected, demonstrating the superiority of EY/Cu dual catalysis. Interestingly, for the highest concentration of the CTA (6.0 mM, DP_T_ = 50), no OEOMA_500_ conversion was observed (entry 1, Table 3). This could be explained by the insufficient loading of the photoredox catalyst. In contrast, under the same conditions, photo-ATRP reached 74% monomer conversion and low dispersity (*M*_n,abs_ = 16 500, *Đ* = 1.22, entry 1, Table 1).

**Table 3:** EY-catalyzed PET-RAFT polymerization of OEOMA_500_ with varying degrees of polymerization^a^

No.	[OEOMA_500_]/[CPADB]/[EYH_2_]/[TEOA]	[CPADB] (mM)	Conv.^b^ (%)	*M*_n,th_	*M*_n,abs_ c	*M*_n,app_ d	*Đ* d
1	50/1/0.0025/0.15	6.0	0	—	—	—	—
2	100/1/0.005/0.3	3.0	53	28 000	32 000	29 500	1.14
3	200/1/0.01/0.6	1.5	75	75 000	82 500	64 000	1.42
4	400/1/0.02/1.2	0.75	58	116 000	98 500	74 000	1.68
5	600/1/0.03/1.8	0.5	50	150 000	108 000	80 000	2.52
6	800/1/0.04/2.4	0.325	51	204 000	98 500	74 000	3.63
7	1000/1/0.05/3.0	0.3	50	250 000	115 000	84 000	4.01

a Reaction conditions [OEOMA_500_] = 300 mM, [CPADB] = 6.0–0.3 mM, [EYH_2_] = 15 μM, [TEOA] = 0.9 mM in PBS with DMSO (10% v/v), irradiated for 30 min under green LEDs (520 nm, 9.0 mW cm^−2^) in an open vial with stirring at 500 rpm.

b Monomer conversion was determined by using ^1^H NMR spectroscopy.

c Molecular weight (*M*_n,abs_) was determined by Mark–Houwink calibration (see ESI).

d Apparent molecular weight (*M*_n,app_) and dispersity (*Đ*) were determined by GPC analysis (DMF as eluent) calibrated to poly(methyl methacrylate) standard.

### Temporal control of green-light-driven ATRP

The EY/Cu dual catalytic system exhibited a high degree of temporal control, as demonstrated by switching the light on/off (Fig. 4C).^87^ Polymerization proceeded only under green light irradiation. No monomer conversion was observed after the light was turned off. Stirring accelerated the diffusion of oxygen into the reaction mixture, facilitating the oxidation of the activator (Cu^I^/L) to its inactive form (Cu^II^/L), while turning off the light prevented its regeneration. This explains the immediate inhibition of open-to-air ATRP when the light is turned off as the Cu(i) species react with oxygen, halting the activation and subsequent propagation process. A high degree of temporal control is usually achieved by using a copper catalyst at a very low concentration, but this results in much broader molar mass distributions.^88,89^

### Block copolymerization

To confirm chain-end fidelity, a chain extension experiment was performed (Fig. 4D). The macroinitiator p(OEOMA_500_) was synthesized with [OEOMA_500_]/[HOBiB]/[EYH_2_]/[CuBr_2_]/[TPMA] molar ratios of 50/1/0.0025/0.05/0.15 (conv. = 70%, *M*_n,app_ = 16 500, *Đ* = 1.20). A sample was then taken from the post-polymerization mixture and used without further purification to prepare an ATRP “cocktail” with OEOMA_300_ monomer (DP_T_ = 250). After 30 min of green light irradiation, the monomer conversion was 70%. SEC analysis showed a clear shift toward higher molecular weights without any shoulder or tailing at lower molecular weights (*M*_n,app_ = 51 000, *Đ* = 1.18), indicating well-controlled polymerization and high retention of chain-end fidelity.

### Low volume polymerization

Conducting RDRP in low volume opens avenues for many important applications,^90–92^ such as high-throughput combinatorial synthesis.^93–97^ However, the use of external deoxygenation methods on a small scale can lead to the loss of volatile substrates. Oxygen tolerance eliminates the need for degassing before polymerization, facilitating the synthesis of polymers in a small volume.

To investigate the low volume performance of the EY/Cu system, a series of reactions at volumes of 250, 150, and 50 μL were performed in open reaction vessels (Table S1†). Polymerizations were carried out with [OEOMA_500_]/[HOBiB]/[EYH_2_]/[CuBr_2_]/[TPMA] molar ratios of 1000/1/0.05/1/3. Despite the reduced volume and target DP of 1000, high monomer conversions (72–80%) were achieved in all reactions, and only for a volume of 50 μL the polymerization was less controlled (*Đ* = 1.46, entry 4, Table S1†).

These results indicate the great potential of this technique for high-throughput screening applications.

### Synthesis of biohybrids

Functional proteins are often inherently unstable and prone to aggregation, which significantly hinders their practical applications. Anchoring polymers to proteins protects protein–polymer hybrids from denaturation, delays their clearance from the body, and reduces the immunological response toward them.^98^ ATRP is very useful for the preparation of polymer–protein conjugates,^45,99–106^ but degassing prior to polymerization can trigger aggregation.^107^

The dual EY/Cu system was applied in protein modifications. First, ATRP initiators were covalently attached to the accessible lysine residues in bovine serum albumin (BSA) to yield the protein macroinitiator BSA-[iBBr]_22_. The model protein–polymer bioconjugate was then prepared by grafting polymer chains from the surface of BSA using molar ratios of [OEOMA_500_]/[BSA-iBBr_22_]/[EYH_2_]/[CuBr_2_]/[TPMA] = 400/0.045/0.02/0.4/0.8 (Fig. 4E). Within 25 minutes, 45% monomer conversion was reached. SEC with multi-angle light scattering (MALS) analysis confirmed the well-controlled synthesis of the protein–polymer hybrid (*M*_n,th_ = 1 848 000, *M*_n,MALS_ = 1 864 000, *Đ* = 1.28, Fig. S3†).

Nucleic acid–polymer conjugates represent another important class of biohybrids used as multifunctional biomaterials in nanoscience and biomedicine.^91,104,108–110^ Therefore, we decided to use the EY/Cu technique to graft polymers from DNA. A 23-mer DNA-based macroinitiator (DNA-iBBr) with *α*-bromoisobutyrate group at the 5′-end was prepared in a DNA synthesizer^111^ and then extended with OEOMA_500_, and molar ratios of [OEOMA_500_]/[DNA-iBBr]/[EYH_2_]/[CuBr_2_]/[TPMA] = 600/1/0.03/0.6/1.8 at a low reaction volume of 250 μL (Fig. 4F). After 30 min of green light irradiation, 76% monomer conversion was achieved. SEC-MALS analysis showed that the DNA-polymer biohybrid had a dispersity of 1.04, and absolute molecular weight close to the theoretical value (*M*_n,th_ = 231 000, *M*_n_,_MALS_ = 259 500, Fig. S4†), indicating a well-controlled polymerization.

### ATRP in the presence of cells

Engineering cell surfaces with synthetic polymers enables the modulation of physicochemical and biological properties of cells.^112^ Both ATRP and RAFT techniques have been used to initiate polymers from the surface of living cells.^80,113^ We wanted to investigate whether our method could also be used for grafting polymers from living cells. For this purpose, the method must allow rapid polymerization in water at low temperature to minimize cell exposure to a potentially harmful polymerization environment and tolerate oxygen since degassing procedures such as freeze–pump–thaw cycles cannot be applied to living cells. In addition, the polymerization process must not cause cell death.

It is a common belief that Cu-catalyzed ATRP methods are more cytotoxic than metal-free RAFT techniques. The cytocompatibility of EY/Cu dual catalysis was investigated and compared with EY-catalyzed PET-RAFT (Fig. 4G). Polymerizations were performed in the presence of human embryonic kidney 293 (HEK293) cells at a low volume of 250 μL using a 96-Well LED array (520 nm, 25 mW cm^−2^). The cells tolerated both the ATRP and RAFT polymerization *in vitro* at a similar level. Compared to the control (untreated cells), 81% of the cells exposed to photo-ATRP for 10 min remained viable *vs.* 85% in PET-RAFT, indicating that the EY/Cu-catalyzed ATRP technique can be utilized in live cell surface engineering.

### Green light-induced ATRP in organic solvents

Next, we expanded the scope of the EY/Cu system to the hydrophobic methyl acrylate (MA) monomer. Polymerizations of MA with a target DP of 200 were performed using EYH_2_, the CuBr_2_/Me_6_TREN complex (Me_6_TREN = tris[2-(dimethylamino)ethyl]amine) and methyl *α*-bromoisobutyrate (MBiB) initiator in DMSO in open vials without stirring (Table 3). The initial conditions used [MA]/[MBiB]/[EYH_2_]/[CuBr_2_]/[Me_6_TREN] molar ratios of 200/1/0.01/0.05/0.3 and MA monomer concentration of 5.5 M. After 60 min of green light irradiation (520 nm, 9.0 mW cm^−2^), the monomer conversion was 48% (entry 1, Table 4) and SEC analysis showed that the polymer had a bimodal molecular weight distribution (Fig. S6A†). This could be attributed to the over-reduction of the Br–Cu^II^/L deactivator by EY, leading to termination by radical–radical coupling. To counter this problem, the EYH_2_ loading was reduced 2-fold (entry 2, Table 4). As a result, a much higher monomer conversion was reached (81%), yielding a well-defined polymer with monomodal, narrow molecular weight distribution (*Đ* = 1.05) and controlled molecular weight (*M*_n,th_ = 14 100, *M*_n,abs_ = 14 300). Further reduction of the amount of EYH_2_ improved monomer conversion (84%), but formed polymer with the dispersity of 1.07 and deviation from the theoretical molecular weight value (*M*_n,th_ = 14 600, *M*_n,abs_ = 12 200). Despite reducing CuBr_2_ to just 100 ppm (relative to monomer), the ATRP was well-controlled (*Đ* = 1.08). Importantly, reducing EYH_2_ 5-fold to just 1 ppm still allowed polymerization of MA (entry 5, Table 4). However, the monomer conversion dropped to 44%, and the dispersity increased to 1.23.

**Table 4:** EY/Cu mediated photo-ATRP of MA^a^

No.	[EYH_2_] (equiv)	CuBr_2_ (equiv)	Me_6_TREN (equiv)	Conv.^b^ (%)	*M*_n,th_	*M*_n,abs_ c	*M*_n,app_ d	*Đ* d
1	0.01	0.05	0.3	48	8500	17 200	21 000	1.12
2	0.005	0.05	0.3	81	14 100	14 300	17 300	1.05
3	0.001	0.05	0.3	84	14 600	12 200	14 600	1.07
4	0.001	0.02	0.12	79	13 800	11 500	13 700	1.08
5	0.0002	0.02	0.12	44	7800	8300	9800	1.23

a Reaction conditions: [MA]/[MBiB]/[EYH_2_]/[CuBr_2_]/[Me_6_TREN] = 200/1/x/x/x, [MA] = 5.5 M, in DMSO, irradiated for 60 min under green LEDs (520 nm, 9.0 mW cm^−2^) in an open vial with no stirring.

b Monomer conversion was determined by using ^1^H NMR spectroscopy.

c Molecular weight (*M*_n,abs_) was determined by Mark–Houwink calibration.

d Apparent molecular weight (*M*_n,app_) and dispersity (*Đ*) were determined by GPC analysis (THF as eluent) calibrated to poly(methyl methacrylate) standards.

### Oxygen concentration measurements

**Fig. 5:**
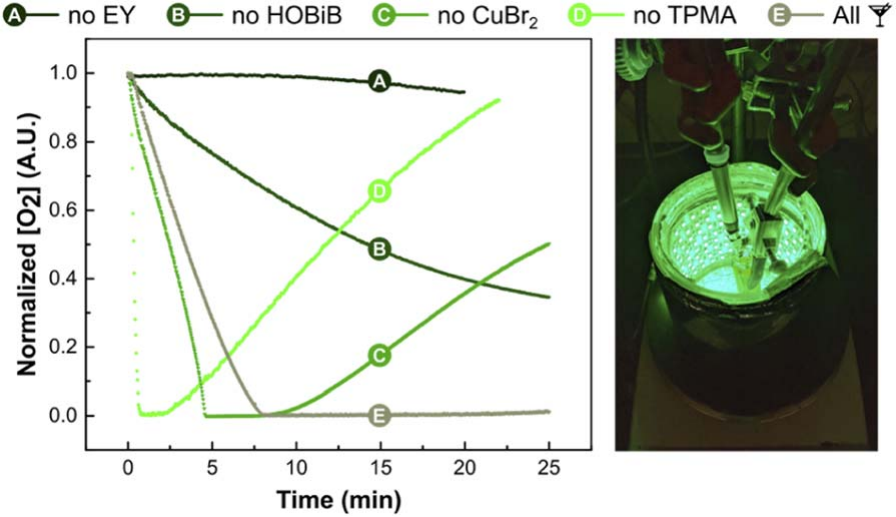
Oxygen concentration measurements. Reactions conditions: [HOBiB]/[EYH_2_]/[CuBr_2_]/[TPMA] = 1/0.01/0.2/0.6, [HOBiB] = 1.5 mM, in PBS with DMSO (10% v/v), irradiated for 30 min under green LEDs (520 nm, 9.0 mW cm^−2^) in an open vial with stirring at 500 rpm.

Finally, the effect of EY/Cu catalysis on oxygen consumption rate was investigated *in situ* using an oxygen probe (Fig. 5).^114^ Measurements were carried out without monomer in PBS with DMSO (10% v/v) in open vials at a stirring rate of 500 rpm. The initial conditions used [HOBiB]/[EYH_2_]/[CuBr_2_]/[TPMA] molar ratios of 1/0.01/0.2/0.6. After ∼7 min of green light irradiation, all dissolved oxygen was scavenged, which correlates well with the induction period observed during kinetic studies (Fig. 3A). Despite the continuous diffusion of oxygen into the reaction mixture, no oxygen re-saturation was observed. As expected, oxygen depletion did not occur in the absence of EY, whereas exclusion of the HOBiB initiator resulted in slower oxygen removal. The Cu-free system initially showed faster oxygen consumption, but after a short time, the oxygen concentration started to increase again. This could be attributed to the photobleaching of EY caused by reactive oxygen species. The Cu/EY catalysis without TPMA ligand showed an even faster initial drop of oxygen concentration followed by a rapid increase. Presumably, in the absence of the ligand, the oxidized EY could not be regenerated, closing the catalytic cycle. The control experiments showed that all components of EY/Cu catalysis are critical to achieving high oxygen tolerance.

## Conclusions

We have developed a highly efficient, biocompatible dual photoredox/copper catalytic system enabling ATRP under low-energy green light irradiation. Eosin Y was used as an organic photocatalyst to trigger and drive the polymerization, while the copper catalyst provided control over the radical propagation *via* ATRP equilibrium. Excited EY was oxidatively quenched by X–Cu^II^/L, generating Cu^I^/L activator and EY˙^+^. The ATRP ligand used in excess then reduced the EY˙^+^, closing the photocatalytic cycle. The technique showed high oxygen tolerance, allowing ATRP in an open vessel at stirring rates up to 1000 rpm. Well-defined polymers (1.15 < *Đ* < 1.22) were synthesized with high OEOMA_500_ monomer conversions for a wide targeted DP range (50–1000) within 30 min under biologically relevant conditions. This contrasts with PET-RAFT polymerization, which under the same conditions led to poorer molecular weight control and higher dispersity values (1.14 < *Đ* < 4.01). Moreover, the photo-ATRP could be stopped immediately and then resumed by turning the green light off and on. The method proved to be highly efficient in the synthesis of protein–polymer and DNA-polymer hybrids without the need for deoxygenation showing similar cytocompatibility to PET-RAFT polymerization. It also enabled the successful open-to-air polymerization of MA monomer in DMSO using Cu and eosin Y catalyst at ppm levels, which extends the scope of this method toward hydrophobic monomers. We envision that this method will provide non-experts with easy access to advanced materials and polymer biohybrids.

## Data availability

Additional data and detailed experimental details are available in the ESI.†

## Author contributions

The manuscript was written through contributions of all authors.

## Conflicts of interest

There are no conflicts to declare.

## Supplementary Material

SC-013-D2SC04210J-s001

## References

[cit1] Gomollón-Bel F. (2019). Chem. Int..

[cit2] Truong N. P., Jones G. R., Bradford K. G. E., Konkolewicz D., Anastasaki A. (2021). Nat. Rev. Chem..

[cit3] Corrigan N., Jung K., Moad G., Hawker C. J., Matyjaszewski K., Boyer C. (2020). Prog. Polym. Sci..

[cit4] Parkatzidis K., Wang H. S., Truong N. P., Anastasaki A. (2020). Chem.

[cit5] Matyjaszewski K., Tsarevsky N. V. (2014). J. Am. Chem. Soc..

[cit6] Matyjaszewski K., Tsarevsky N. V. (2009). Nat. Chem..

[cit7] Tsarevsky N. V., Matyjaszewski K. (2007). Chem. Rev..

[cit8] Ribelli T. G., Lorandi F., Fantin M., Matyjaszewski K. (2019). Macromol. Rapid Commun..

[cit9] Enciso A. E., Lorandi F., Mehmood A., Fantin M., Szczepaniak G., Janesko B. G., Matyjaszewski K. (2020). Angew. Chem., Int. Ed..

[cit10] Matyjaszewski K. (2012). Macromolecules.

[cit11] Lorandi F., Fantin M., Matyjaszewski K. (2022). J. Am. Chem. Soc..

[cit12] Fung A. K. K., Coote M. L. (2021). Polym. Int..

[cit13] Wang J.-S., Matyjaszewski K. (1995). J. Am. Chem. Soc..

[cit14] Szczepaniak G., Fu L., Jafari H., Kapil K., Matyjaszewski K. (2021). Acc. Chem. Res..

[cit15] Fromel M., Benetti E. M., Pester C. W. (2022). ACS Macro Lett..

[cit16] Yeow J., Chapman R., Gormley A. J., Boyer C. (2018). Chem. Soc. Rev..

[cit17] Matyjaszewski K., Coca S., Gaynor S. G., Wei M., Woodworth B. E. (1998). Macromolecules.

[cit18] Min K., Jakubowski W., Matyjaszewski K. (2006). Macromol. Rapid Commun..

[cit19] Matyjaszewski K., Dong H., Jakubowski W., Pietrasik J., Kusumo A. (2007). Langmuir.

[cit20] De Bon F., Fonseca R. G., Lorandi F., Serra A. C., Isse A. A., Matyjaszewski K., Coelho J. F. J. (2022). Chem. Eng. J..

[cit21] Li W., Sheng W., Li B., Jordan R. (2021). Angew. Chem., Int. Ed..

[cit22] Liarou E., Whitfield R., Anastasaki A., Engelis N. G., Jones G. R., Velonia K., Haddleton D. M. (2018). Angew. Chem., Int. Ed..

[cit23] Li R., Kong W., An Z. (2022). Angew. Chem., Int. Ed..

[cit24] Navarro L. A., Enciso A. E., Matyjaszewski K., Zauscher S. (2019). J. Am. Chem. Soc..

[cit25] Enciso A. E., Fu L., Russell A. J., Matyjaszewski K. (2018). Angew. Chem., Int. Ed..

[cit26] Enciso A. E., Fu L., Lathwal S., Olszewski M., Wang Z., Das S. R., Russell A. J., Matyjaszewski K. (2018). Angew. Chem., Int. Ed..

[cit27] Wei Q., Sun M., Lorandi F., Yin R., Yan J., Liu T., Kowalewski T., Matyjaszewski K. (2021). Macromolecules.

[cit28] Yan W., Dadashi-Silab S., Matyjaszewski K., Spencer N. D., Benetti E. M. (2020). Macromolecules.

[cit29] Dadashi-Silab S., Szczepaniak G., Lathwal S., Matyjaszewski K. (2020). Polym. Chem..

[cit30] Dadashi-Silab S., Pan X., Matyjaszewski K. (2017). Macromolecules.

[cit31] Mosnáček J., Eckstein-Andicsová A., Borská K. (2015). Polym. Chem..

[cit32] Szczepaniak G., Łagodzińska M., Dadashi-Silab S., Gorczyński A., Matyjaszewski K. (2020). Chem. Sci..

[cit33] Qiao L., Zhou M., Shi G., Cui Z., Zhang X., Fu P., Liu M., Qiao X., He Y., Pang X. (2022). J. Am. Chem. Soc..

[cit34] Liarou E., Han Y., Sanchez A. M., Walker M., Haddleton D. M. (2020). Chem. Sci..

[cit35] Fan G., Graham A. J., Kolli J., Lynd N. A., Keitz B. K. (2020). Nat. Chem..

[cit36] Kang H., Jeong W., Hong D. (2019). Langmuir.

[cit37] Dunderdale G. J., Urata C., Miranda D. F., Hozumi A. (2014). ACS Appl. Mater. Interfaces.

[cit38] Aydogan C., Yilmaz G., Shegiwal A., Haddleton D. M., Yagci Y. (2022). Angew. Chem., Int. Ed..

[cit39] Pan X., Tasdelen M. A., Laun J., Junkers T., Yagci Y., Matyjaszewski K. (2016). Prog. Polym. Sci..

[cit40] Dadashi-Silab S., Doran S., Yagci Y. (2016). Chem. Rev..

[cit41] Chen M., Zhong M., Johnson J. A. (2016). Chem. Rev..

[cit42] Ribelli T. G., Konkolewicz D., Bernhard S., Matyjaszewski K. (2014). J. Am. Chem. Soc..

[cit43] Mosnáček J., Ilčíková M. (2012). Macromolecules.

[cit44] Tasdelen M. A., Uygun M., Yagci Y. (2011). Macromol. Rapid Commun..

[cit45] Olson R. A., Korpusik A. B., Sumerlin B. S. (2020). Chem. Sci..

[cit46] Corbin D. A., Miyake G. M. (2022). Chem. Rev..

[cit47] Discekici E. H., Anastasaki A., Read de Alaniz J., Hawker C. J. (2018). Macromolecules.

[cit48] McCarthy B., Miyake G. M. (2018). ACS Macro Lett..

[cit49] Narupai B., Page Z. A., Treat N. J., McGrath A. J., Pester C. W., Discekici E. H., Dolinski N. D., Meyers G. F., Read de Alaniz J., Hawker C. J. (2018). Angew. Chem., Int. Ed..

[cit50] Theriot Jordan C., Lim C.-H., Yang H., Ryan Matthew D., Musgrave Charles B., Miyake Garret M. (2016). Science.

[cit51] Pan X., Fang C., Fantin M., Malhotra N., So W. Y., Peteanu L. A., Isse A. A., Gennaro A., Liu P., Matyjaszewski K. (2016). J. Am. Chem. Soc..

[cit52] Treat N. J., Sprafke H., Kramer J. W., Clark P. G., Barton B. E., Read de Alaniz J., Fors B. P., Hawker C. J. (2014). J. Am. Chem. Soc..

[cit53] Zhou L., Zhang Z., Li M., Wang Q., Gao J., Li K., Lei L. (2021). Green Chem..

[cit54] Xu X., Xu X., Zeng Y., Zhang F. (2021). J. Photochem. Photobiol. A.

[cit55] Ravetz B. D., Pun A. B., Churchill E. M., Congreve D. N., Rovis T., Campos L. M. (2019). Nature.

[cit56] Kutahya C., Aykac F. S., Yilmaz G., Yagci Y. (2016). Polym. Chem..

[cit57] Averick S., Simakova A., Park S., Konkolewicz D., Magenau A. J. D., Mehl R. A., Matyjaszewski K. (2012). ACS Macro Lett..

[cit58] Dadashi-Silab S., Kim K., Lorandi F., Szczepaniak G., Kramer S., Peteanu L., Matyjaszewski K. (2022). ACS Macro Lett..

[cit59] Dadashi-Silab S., Lorandi F., DiTucci M. J., Sun M., Szczepaniak G., Liu T., Matyjaszewski K. (2021). J. Am. Chem. Soc..

[cit60] Sun M., Lorandi F., Yuan R., Dadashi-Silab S., Kowalewski T., Matyjaszewski K. (2021). Front. Chem..

[cit61] Kütahya C., Zhai Y., Li S., Liu S., Li J., Strehmel V., Chen Z., Strehmel B. (2021). Angew. Chem., Int. Ed..

[cit62] Kütahya C., Meckbach N., Strehmel V., Strehmel B. (2021). J. Polym. Sci..

[cit63] Zhang W., He J., Lv C., Wang Q., Pang X., Matyjaszewski K., Pan X. (2020). Macromolecules.

[cit64] Kütahya C., Schmitz C., Strehmel V., Yagci Y., Strehmel B. (2018). Angew. Chem., Int. Ed..

[cit65] Tasdelen M. A., Ciftci M., Yagci Y. (2012). Macromol. Chem. Phys..

[cit66] Xu J., Jung K., Atme A., Shanmugam S., Boyer C. (2014). J. Am. Chem. Soc..

[cit67] Wu C., Corrigan N., Lim C.-H., Liu W., Miyake G., Boyer C. (2022). Chem. Rev..

[cit68] Wu C., Jung K., Ma Y., Liu W., Boyer C. (2021). Nat. Commun..

[cit69] Allegrezza M. L., Konkolewicz D. (2021). ACS Macro Lett..

[cit70] Shanmugam S., Xu J., Boyer C. (2015). J. Am. Chem. Soc..

[cit71] Shanmugam S., Xu J., Boyer C. (2015). Chem. Sci..

[cit72] Shanmugam S., Xu J., Boyer C. (2016). Angew. Chem., Int. Ed..

[cit73] Xu J., Jung K., Corrigan N. A., Boyer C. (2014). Chem. Sci..

[cit74] Theriot J. C., Miyake G. M., Boyer C. A. (2018). ACS Macro Lett..

[cit75] Wu Z., Fang W., Wu C., Corrigan N., Zhang T., Xu S., Boyer C. (2022). Chem. Sci..

[cit76] Yeow J., Chapman R., Xu J., Boyer C. (2017). Polym. Chem..

[cit77] Lueckerath T., Strauch T., Koynov K., Barner-Kowollik C., Ng D. Y. W., Weil T. (2019). Biomacromolecules.

[cit78] Figg C. A., Hickman J. D., Scheutz G. M., Shanmugam S., Carmean R. N., Tucker B. S., Boyer C., Sumerlin B. S. (2018). Macromolecules.

[cit79] Xu J., Shanmugam S., Duong H. T., Boyer C. (2015). Polym. Chem..

[cit80] Niu J., Lunn D. J., Pusuluri A., Yoo J. I., O’Malley M. A., Mitragotri S., Soh H. T., Hawker C. J. (2017). Nat. Chem..

[cit81] Fantin M., Isse A. A., Gennaro A., Matyjaszewski K. (2015). Macromolecules.

[cit82] Majek M., Jacobi von Wangelin A. (2016). Acc. Chem. Res..

[cit83] Romero N. A., Nicewicz D. A. (2016). Chem. Rev..

[cit84] Martínez-Haya R., Heredia A. A., Castro-Godoy W. D., Schmidt L. C., Marin M. L., Argüello J. E. (2021). J. Org. Chem..

[cit85] Parkatzidis K., Boner S., Wang H. S., Anastasaki A. (2022). ACS Macro Lett..

[cit86] Cuthbert J., Wanasinghe S.
V., Matyjaszewski K., Konkolewicz D. (2021). Macromolecules.

[cit87] Dadashi-Silab S., Lee I.-H., Anastasaki A., Lorandi F., Narupai B., Dolinski N. D., Allegrezza M. L., Fantin M., Konkolewicz D., Hawker C. J., Matyjaszewski K. (2020). Macromolecules.

[cit88] Whitfield R., Parkatzidis K., Rolland M., Truong N. P., Anastasaki A. (2019). Angew. Chem., Int. Ed..

[cit89] Wang H. S., Parkatzidis K., Harrisson S., Truong N. P., Anastasaki A. (2021). Chem. Sci..

[cit90] Ślusarczyk K., Flejszar M., Chmielarz P. (2021). Polymer.

[cit91] Sun Y., Lathwal S., Wang Y., Fu L., Olszewski M., Fantin M., Enciso A. E., Szczepaniak G., Das S., Matyjaszewski K. (2019). ACS Macro Lett..

[cit92] Liarou E., Anastasaki A., Whitfield R., Iacono C. E., Patias G., Engelis N. G., Marathianos A., Jones G. R., Haddleton D. M. (2019). Polym. Chem..

[cit93] Gormley A. J., Webb M. A. (2021). Nat. Rev. Mater..

[cit94] Li Z., Han Z., Stenzel M. H., Chapman R. (2022). Nano Lett..

[cit95] Soheilmoghaddam F., Rumble M., Cooper-White J. (2021). Chem. Rev..

[cit96] Judzewitsch P. R., Corrigan N., Trujillo F., Xu J., Moad G., Hawker C. J., Wong E. H. H., Boyer C. (2020). Macromolecules.

[cit97] Tamasi M., Kosuri S., DiStefano J., Chapman R., Gormley A. J. (2020). Adv. Intell. Syst..

[cit98] Pelegri-O’Day E. M., Lin E.-W., Maynard H. D. (2014). J. Am. Chem. Soc..

[cit99] Olszewski M., Jeong J., Szczepaniak G., Li S., Enciso A., Murata H., Averick S., Kapil K., Das S. R., Matyjaszewski K. (2022). ACS Macro Lett..

[cit100] Theodorou A., Mandriotis P., Anastasaki A., Velonia K. (2021). Polym. Chem..

[cit101] Messina M. S., Messina K. M. M., Bhattacharya A., Montgomery H. R., Maynard H. D. (2020). Prog. Polym. Sci..

[cit102] Theodorou A., Liarou E., Haddleton D. M., Stavrakaki I. G., Skordalidis P., Whitfield R., Anastasaki A., Velonia K. (2020). Nat. Commun..

[cit103] Chen C., Ng D. Y. W., Weil T. (2020). Prog. Polym. Sci..

[cit104] Baker S. L., Kaupbayeva B., Lathwal S., Das S. R., Russell A. J., Matyjaszewski K. (2019). Biomacromolecules.

[cit105] Murata H., Carmali S., Baker S. L., Matyjaszewski K., Russell A. J. (2018). Nat. Commun..

[cit106] Russell A. J., Baker S. L., Colina C. M., Figg C. A., Kaar J. L., Matyjaszewski K., Simakova A., Sumerlin B. S. (2018). AIChE J..

[cit107] Zhang L., Zhang Y., Cheng J., Wang L., Wang X., Zhang M., Gao Y., Hu J., Zhang X., Lü J., Li G., Tai R., Fang H. (2017). Sci. Rep..

[cit108] Whitfield C. J., Zhang M., Winterwerber P., Wu Y., Ng D. Y. W., Weil T. (2021). Chem. Rev..

[cit109] Yerneni S. S., Lathwal S., Cuthbert J., Kapil K., Szczepaniak G., Jeong J., Das S. R., Campbell P. G., Matyjaszewski K. (2022). Biomacromolecules.

[cit110] Lathwal S., Yerneni S. S., Boye S., Muza U. L., Takahashi S., Sugimoto N., Lederer A., Das S. R., Campbell P. G., Matyjaszewski K. (2021). Proc. Natl. Acad. Sci. U. S. A..

[cit111] Pan X., Lathwal S., Mack S., Yan J., Das S. R., Matyjaszewski K. (2017). Angew. Chem., Int. Ed..

[cit112] Liu J., Liu B. (2022). Prog. Polym. Sci..

[cit113] Kim J. Y., Lee B. S., Choi J., Kim B. J., Choi J. Y., Kang S. M., Yang S. H., Choi I. S. (2016). Angew. Chem., Int. Ed..

[cit114] Rolland M., Whitfield R., Messmer D., Parkatzidis K., Truong N. P., Anastasaki A. (2019). ACS Macro Lett..

