# Supplementary material for: Open-air green-light-driven ATRP enabled by dual photoredox/copper catalysis
Source: Chem Sci. 2022 Sep 20;13(39):11540–50. doi: 10.1039/d2sc04210j (PMC9557244; doi:10.1039/d2sc04210j)
Supplement: SC-013-D2SC04210J-s001 [file SC-013-D2SC04210J-s001.pdf]

Electronic Supplementary Material (ESI) for Chemical Science.
This journal is © The Royal Society of Chemistry 2022

Supplementary Information

Open-air green-light-driven ATRP enabled by dual photoredox/copper catalysis

Grzegorz Szczepaniak,*[ab] Jaepil Jeong,[a] Kriti Kapil,[a] Sajjad Dadashi-Silab,[a] Saigopalakrishna S. Yerneni,[c] Paulina Ratajczyk,[ad] Sushil Lathwal,[ae] Dirk J. Schild,[a] Subha R. Das,[ae] and Krzysztof Matyjaszewski*[a]

a) Department of Chemistry, Carnegie Mellon University, 4400 Fifth Avenue, Pittsburgh, PA 15213, United States. E-mail: km3b@andrew.cmu.edu, gszczepa@andrew.cmu.edu
b) Faculty of Chemistry, University of Warsaw, Pasteura 1, 02-093 Warsaw, Poland.
c) Department of Biomedical Engineering and Engineering Research Accelerator, 5000 Forbes Avenue, Carnegie Mellon University, Pittsburgh, PA 15213, United States
d) Faculty of Chemistry, Adam Mickiewicz University, Uniwersytetu Poznańskiego 8, 61-614 Poznań, Poland.
e) The Center for Nucleic Acids Science & Technology, 4400 Fifth Avenue, Carnegie Mellon University, Pittsburgh, PA 15213, United States

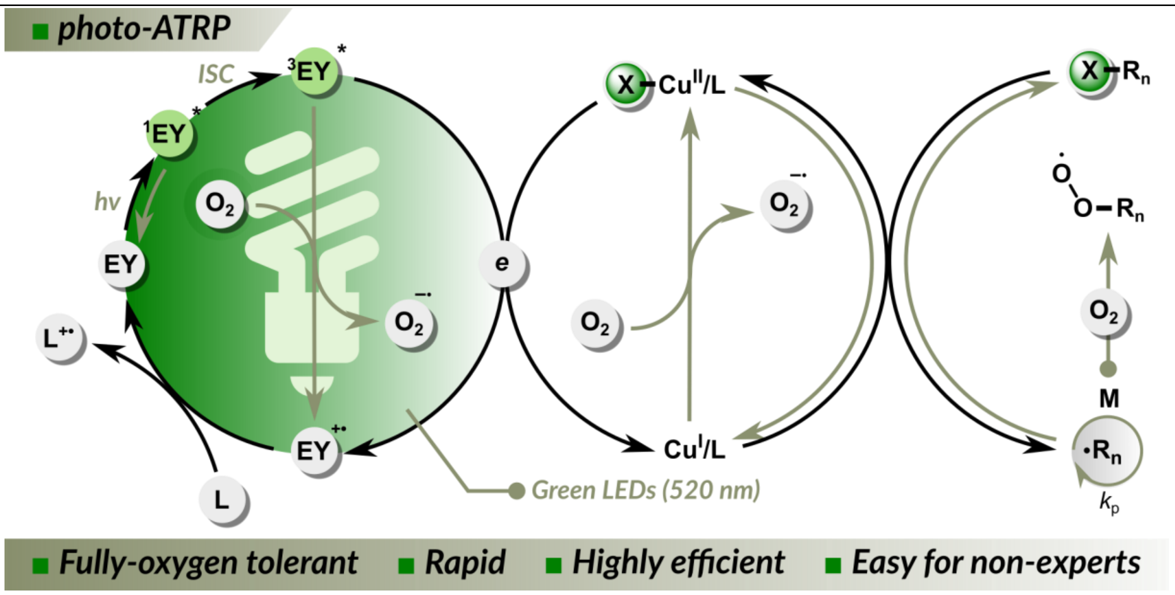

Supplementary Information

Table of contents

Experimental Details 4

Materials 4

Cell Culture 4

Instrumentation 4

Nuclear Magnetic Resonance (NMR) 4

Size Exclusion Chromatography (SEC) 4

Size Exclusion Chromatography with Multi-Angle Light Scattering (SEC-MALS) 5

Fluorescence Quenching Experiments 5

DNA Synthesis 5

Oxygen measurements 5

Polymerizations 5

Procedures 6

General Procedure for EY/Cu-catalyzed ATRP of $OEOMA_{500}$ 6

Fluorescence Quenching Experiments (Figure S3) 8

General Procedure for EY-catalyzed PET-RAFT Polymerization of $OEOMA_{500}$ 8

Kinetics of Green Light-Induced ATRP (Figure 3A) 9

Kinetics of PET-RAFT (Figure 3D) 10

Green Light-Induced ATRP of $OEOMA_{500}$ with Varying Degrees of Polymerization (Table 2) 10

PET-RAFT Polymerization of $OEOMA_{500}$ with Varying Degrees of Polymerization (Table 3) 10

Temporal Control of Green Light-Induced ATRP (Figure 4C) 11

Block Copolymerization (Figure 4D) 11

Low Volume Polymerizations (Table S1) 11

Table S1. Low volume ATRP[a] 12

Synthesis of Protein Macroinitiator ($BSA\text{-}iBB_{22}$) 12

Synthesis of Protein-Polymer Biohybrid (Figure 4E) 13

Supplementary Information

Synthesis of DNA-Polymer Biohybrid (Figure 4F) 13

ATRP in the Presence of Cells – Cytotoxicity Assay (Figure 4G) 14

General Procedure for EY/Cu-catalyzed ATRP of MA in DMSO 16

Determination of Mark-Houwink Parameters of $OEOMA_{500}$ in DMF 17

Table S2. Molecular weight and natural log of PMMA and p($OEOMA_{500}$) with the same elution time.[a] 19

References 21

Supplementary Information

Experimental Details

Materials

All chemicals were purchased from commercial sources and used as received unless otherwise noted. Tris(2-pyridylmethyl)amine (**TPMA**, 99%), tris[2-(dimethylamino)ethyl]amine (**Me_6TREN**, 99%) ware purchased from *AmBeed.* Eosin Y (**EYH_2**, 99%), copper(II) bromide ($CuBr_2$, 99.99%), 2-hydroxyethyl 2-bromoisobutyrate (**HOBiB**, 95%), methyl α-bromoisobutyrate (**MBiB**, ≥99%), 4-cyano-4-(phenylcarbonothioylthio)pentanoic acid (**CPADB**), 1,4-bis(3-isocyanopropyl)piperazine (**QA**), triethanolamine (**TEOA**, ≥99.0%), Aminomethyl *ChemMatrix*® resin (0.50-0.70 mmol/g amine loading) were purchased from *Sigma-Aldrich.* 10X PBS and 10X DPBS solutions ware purchased from *Thermo Fisher Scientific.* Oligo(ethylene glycol) methyl ether methacrylate (average M_n = 500, **$OEOMA_{500}$**) was purchased from *Sigma-Aldrich* and passed through a column of basic alumina to remove inhibitor prior to use. Water (HPLC grade) and dimethyl sulfoxide (DMSO, ≥99.7%) were purchased from *Fisher Chemical.* DNA phosphoramidites and CPG solid support for DNA synthesis ware purchased from *Chemgenes* and *Glen Research.*

Cell Culture

Human embryonic kidney cells (HEK293; ATCC® CRL-1573™, Manassas) were grown and maintained in Dulbecco's modified eagle media (DMEM; *Gibco)* supplemented with 10% fetal bovine serum (*Thermo Fisher Scientific*) and 1% Penicillin-streptomycin (*Gibco*).

Instrumentation

Nuclear Magnetic Resonance (NMR)

^{1}H NMR spectra were recorded on *Bruker* Avance III 500 MHz spectrometers with D_2O or DMSO-d6 used as the solvent.

Size Exclusion Chromatography (SEC)

SEC measurements of p($OEOMA_{500}$) were performed using PSS columns (Styrogel 10^5 , 10^3 , 10^2 Å) with DMF as an eluent at 50 °C and the flow rate of 1 mL/min. Linear poly(methyl methacrylate) standards were used for calibration. SEC measurements of poly(methyl acrylate) were conducted using PSS columns (Styrogel 10^2, 10^3, 10^4, 10^5 Å) with THF as an eluent at 35 °C and the flow rate of 1 mL/min. Linear poly(methyl methacrylate) standards were used for calibration.

Supplementary Information

Size Exclusion Chromatography with Multi-Angle Light Scattering (SEC-MALS)

SEC-MALS measurements of polymers and bioconjugates were performed using Agilent SEC system (*Agilent*, 1260 Infinity II with UV detector) coupled with MALS, DLS, Viscometer and RI detectors (*Wyatt Technology*, USA). Measurements were performed using *Waters* Ultrahydrogel Linear column with 1X DPBS as an eluent at rt and the flow rate of 0.5 mL/min.

Fluorescence Quenching Experiments

Emission measurements were performed in a four-window 1x1 cm path length quartz microcuvette (*ThorLabs*) using a Edinburgh Instruments FS5 spectrophotometer.

DNA Synthesis

The model DNA initiator (T10-iBBr) was synthesized following the previously reported protocol in the *MerMade 4* oligonucleotide synthesizer (*Bioautomation*).[1]

Oxygen measurements

Oxygen measurements were performed using *FireStingGO$_2$* pocket oxygen meter and a solvent-resistant oxygen probe purchased from *PyroScience.*

Polymerizations

Polymerizations were carried out in open-to-air vials under green LEDs purchased from *aspectLED* (520 nm, 9.0 mW/cm^2). The LED strips were mounted inside a glass container (diameter = 9 cm, height = 7 cm). Polymerizations in the presence of cells were carried out in a 96-well plate using the *Lumidox 96-Well Green LED Array* (520 nm, 20.0 mW/cm^2).

Procedures

General Procedure for EY/Cu-catalyzed ATRP of $OEOMA_{500}$

Prior to polymerization, stock solutions of HOBiB (15.8 mg in 1.0 mL DMSO), $CuBr_2$ (33.5 mg in 20.0 mL DMSO), TPMA (13.1 mg in 1.0 mL DMSO) and EYH_2 (9.7 mg in 10 mL DMSO) were prepared (Figure S1A). The ATRP cocktail was then prepared as follows. In a 5 mL volumetric flask, 750 mg of $OEOMA_{500}$ was weighed (Figure S1B). $CuBr_2$ stock (200 µL), TPMA stock (100 µL), HOBiB stock (100 µL), EYH_2 stock (50 µL), DMSO (50 µL) and 10X PBS solution (500 µL) were then added (Figure S1C). Finally, water was added to the mark on the volumetric flask, and the reaction mixture was stirred on a vortex (Figure S1D). The final concentrations were $OEOMA_{500}$ (300 mM), HOBiB (1.5 mM), EYH_2 (15 µM), $CuBr_2$ (0.3 mM), TPMA (0.9 mM), DMSO (10% v/v). Then 4.4 mL of the ATRP cocktail was added to a 1 dram (12/96 mm) vial equipped with a magnetic stirrer (Figure S1E). The polymerization mixture was stirred at 500 rpm for 30 min under green LEDs (520 nm, 9.0 mW/cm^2) (Figure S1F). Samples were taken and analyzed by ^{1}H NMR and SEC techniques (Figure S1G).

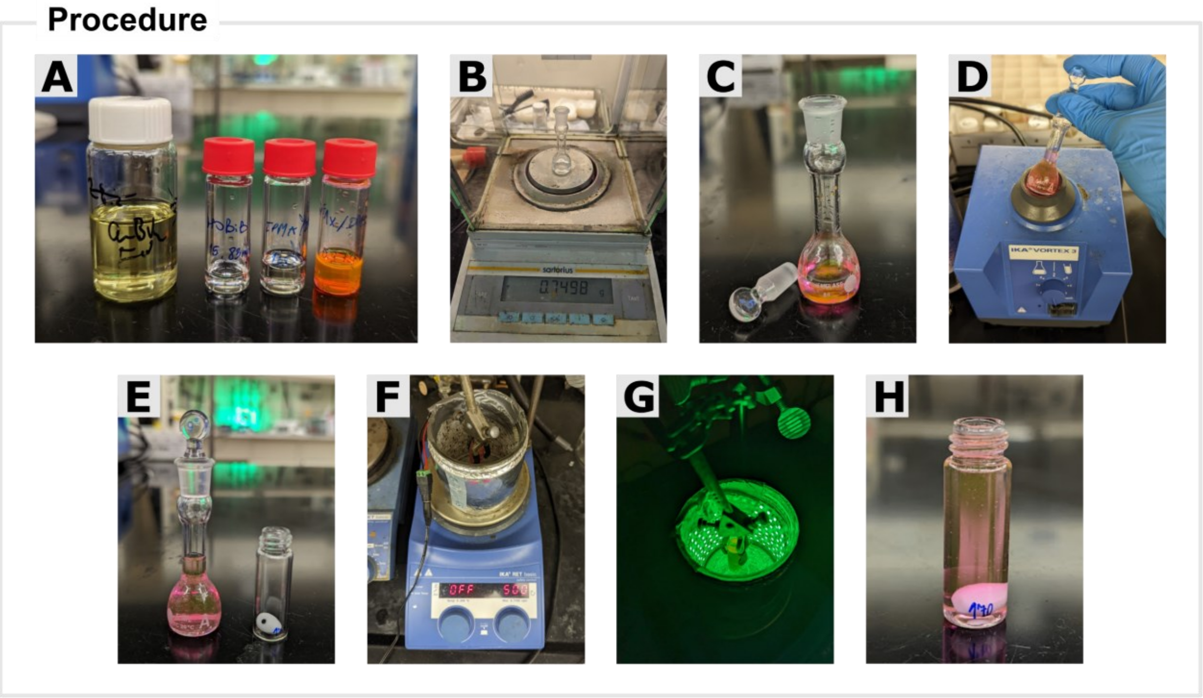

Figure S1. Reaction set-up for EY/Cu-catalyzed ATRP of $OEOMA_{500}$.

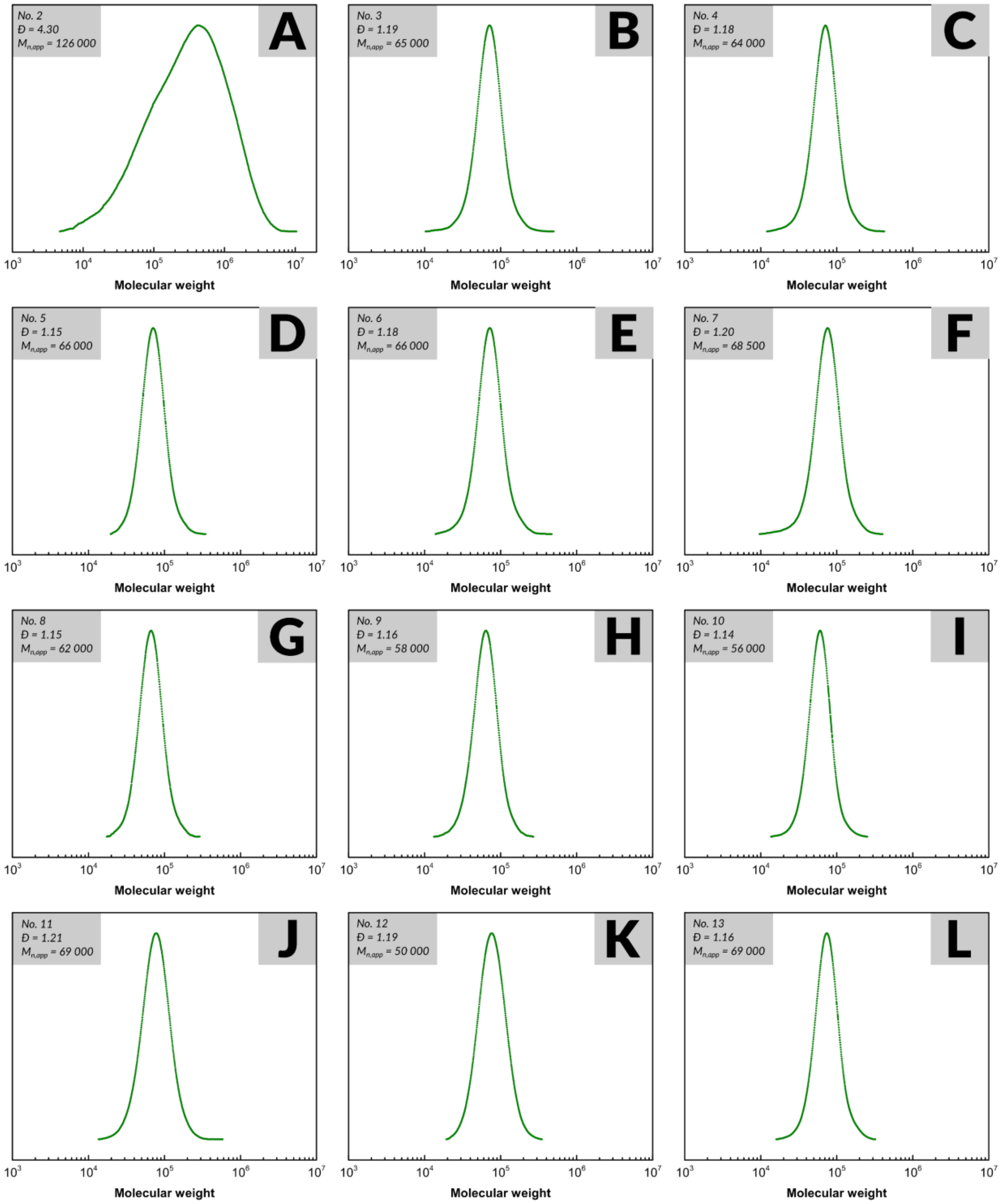

Figure S2. GPC traces for the polymerizations from Table 1.

Fluorescence Quenching Experiments (Figure S3)

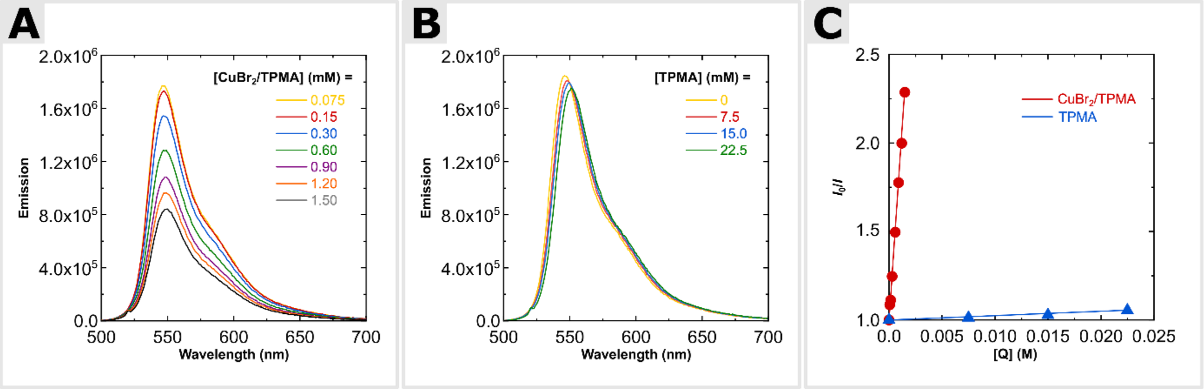

Figure S3. Emission spectra of EY excited at 520 nm upon addition of different concentrations of (A) $CuBr_2$/TPMA (1/1); and (B) TPMA as excited EY quenchers. A decrease in the intensity of the emission spectra at ~550 nm can be related to the excited EY quenching with $CuBr_2$/TPMA and slightly with TPMA. (C) The Stern–Volmer plot for EY (15 µM) in the presence of $CuBr_2$/TPMA or TPMA as quenchers (Q) in PBS with DMSO (10% *v*/*v*) (non-deoxygenated) showing fluorescence quenching mainly by the Cu catalyst.

General Procedure for EY-catalyzed PET-RAFT Polymerization of $OEOMA_{500}$

Prior to polymerization, stock solutions of CPADB (8.4 mg in 2.0 mL DMSO), TEOA (13.4 mg in 2.0 mL DMSO) and EYH_2 (9.7 mg in 10 mL DMSO) were prepared. The RAFT cocktail was then prepared as follows. In a 5 mL volumetric flask, 750 mg of $OEOMA_{500}$ was weighed. TEOA stock (100 µL), CPADB stock (100 µL), EYH_2 stock (50 µL), DMSO (250 µL) and 10X PBS solution (500 µL) were then added. Finally, water was added to the mark on the volumetric flask, and the reaction mixture was stirred on a vortex. The final concentrations were $OEOMA_{500}$ (300 mM), CPADB (1.5 mM), EYH_2 (15 µM), TEOA (0.9 mM), DMSO (10% v/v). Then 4.4 mL of the RAFT cocktail was added to a 1 dram (12/96 mm) vial equipped with a magnetic stirrer. The polymerization mixture was stirred at 500 rpm for 30 min under green LEDs (520 nm, 9.0 mW/cm^2). Samples were taken and analyzed by ^{1}H NMR and SEC techniques.

Kinetics of Green Light-Induced ATRP (Figure 3A)

The ATRP "cocktail" (5 mL) was prepared according to the general procedure. The final concentrations were $OEOMA_{500}$ (300 mM), HOBiB (1.5 mM), EYH_2 (15 µM), $CuBr_2$ (0.3 mM), TPMA (0.9 mM), DMSO (10% v/v). Then 4.4 mL of the ATRP cocktail was added to a 1 dram (12/96 mm) vial equipped with a magnetic stirrer. The polymerization mixture was stirred at 500 rpm for 30 min under green LEDs (520 nm, 9.0 mW/cm^2). Samples were taken at various time intervals to monitor by ^{1}H NMR (D_2O), the decrease in [$OEOMA_{500}$] *vs.* time.

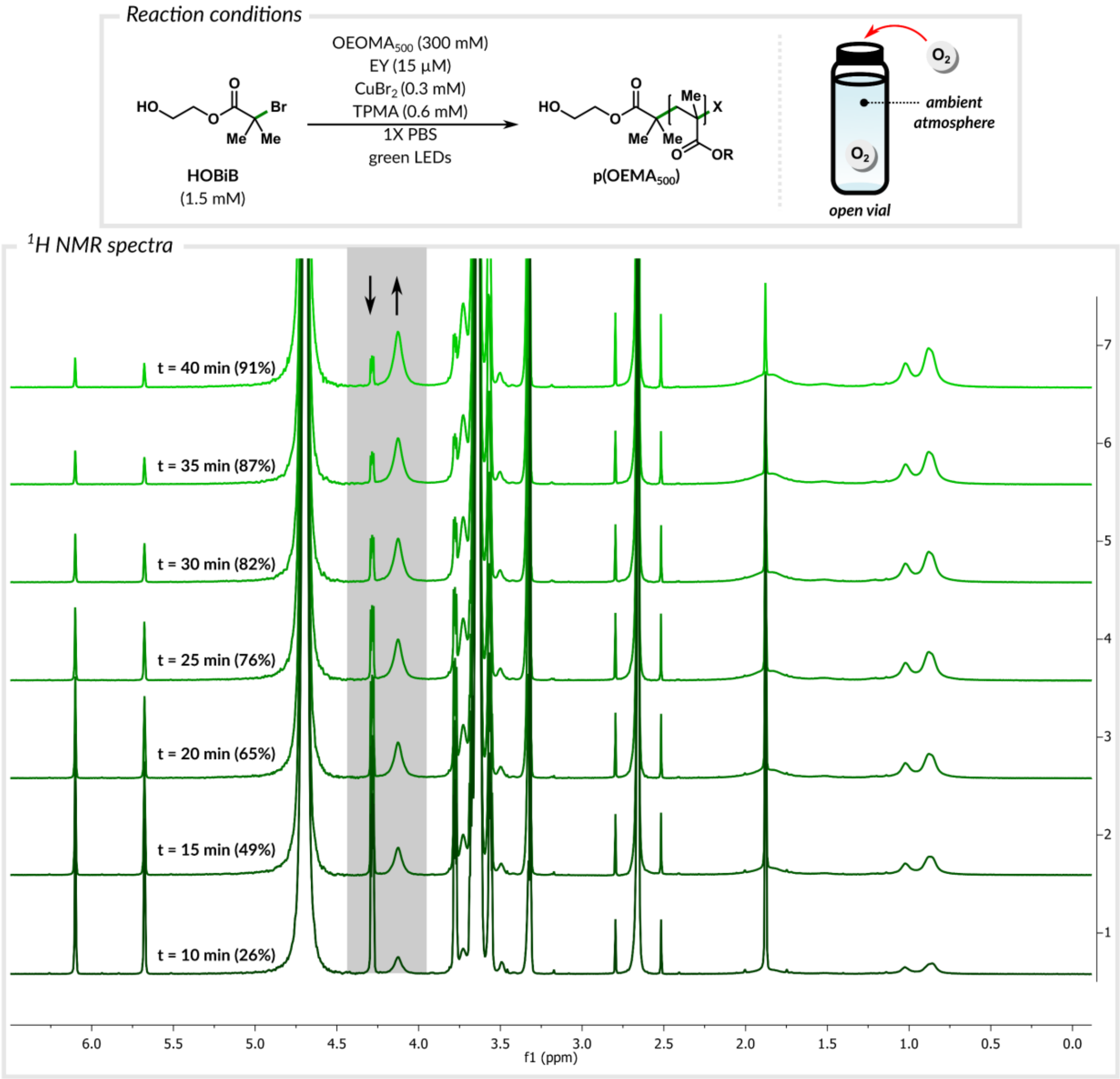

Figure S4. Kinetics of photo-ATRP.

Supplementary Information

Kinetics of PET-RAFT (Figure 3D)

The RAFT “cocktail” (5 mL) was prepared according to the general procedure. The final concentrations were $OEOMA_{500}$ (300 mM), CPADB (1.5 mM), EYH_2 (15 μM), TEOA (0.9 mM), DMSO (10% v/v). Then 4.4 mL of the RAFT cocktail was added to a 1 dram (12/96 mm) vial equipped with a magnetic stirrer. The polymerization mixture was stirred at 500 rpm for 30 min under green LEDs (520 nm, 9.0 mW/cm^2). Samples were taken at various time intervals to monitor by ^{1}H NMR (D_2O), the decrease in [$OEOMA_{500}$] *vs.* time.

Green Light-Induced ATRP of $OEOMA_{500}$ with Varying Degrees of Polymerization (Table 2)

The target degrees of polymerization (DP_T) were varied by adjusting the HOBiB concentration (0.3–0.6 mM), while the concentrations of $OEOMA_{500}$ (300 mM), EYH_2 (15 μM), $CuBr_2$ (0.3 mM), TPMA (0.9 mM), and DMSO (10% v/v) were fixed for each reaction. HOBiB stock solutions of different concentrations (150, 75, 32.5, 25, 18.8, or 15 mM) were prepared by diluting the HOBiB solution (300 mM, 63.3 mg in 1.0 mL DMSO) with DMSO. ATRP cocktails (5 mL) were prepared according to the general procedure by adding HOBiB stock solution (100 μL) at a specific concentration (300, 150, 75, 32.5, 25, 18.8, or 15 mM) to obtain the target DP (DP_T =50, 100, 200, 400, 600, or 1000, respectively). Then 4.4 mL of the ATRP cocktail with a target DP was added to a 1 dram (12/96 mm) vial equipped with a magnetic stirrer. The polymerization mixture was stirred at 500 rpm for 30 min under green LEDs (520 nm, 9.0 mW/cm^2). Samples were taken and analyzed by ^{1}H NMR and SEC techniques.

PET-RAFT Polymerization of $OEOMA_{500}$ with Varying Degrees of Polymerization (Table 3)

The target degrees of polymerization (DP_T) were varied by adjusting the CPADB concentration (0.3–0.6 mM), while the concentrations of $OEOMA_{500}$ (300 mM), EYH_2 (15 μM), TEOA (0.9 mM), and DMSO (10% v/v) were fixed for each reaction. CPADB stock solutions of different concentrations (150, 75, 32.5, 25, 18.8, or 15 mM) were prepared by diluting the CPADB solution (300 mM, 63.3 mg in 1.0 mL DMSO) with DMSO. RAFT cocktails (5 mL) were prepared according to the general procedure by adding CPADB stock solution (100 μL) at a specific concentration (300, 150, 75, 32.5, 25, 18.8, or 15 mM) to obtain the target DP (DP_T =50, 100, 200, 400, 600, or 1000, respectively). Then 4.4 mL of the RAFT cocktail with a target DP was added to a 1 dram (12/96 mm) vial equipped with a magnetic stirrer. The polymerization

mixture was stirred at 500 rpm for 30 min under green LEDs (520 nm, 9.0 mW/cm^2). Samples were taken and analyzed by ^{1}H NMR and SEC techniques.

Temporal Control of Green Light-Induced ATRP (Figure 4C)

The ATRP cocktail (5 mL) was prepared according to the general procedure. The final concentrations were OEOMA$_{500}$ (300 mM), HOBiB (1.5 mM), EYH$_2$ (15 μM), CuBr$_2$ (0.3 mM), TPMA (0.9 mM), DMSO (10% v/v). Then 4.4 mL of the ATRP cocktail was added to a 1 dram (12/96 mm) vial equipped with a magnetic stirrer. The polymerization mixture was stirred at 500 rpm under green LEDs (520 nm, 9.0 mW/cm^2) turning the light *on*/*off* at 15 min intervals. Samples (50 μL) were taken and quenched with 20 μL of 1,4-bis(3-isocyanopropyl)piperazine (10 mg/mL in D_2O) and then analyzed by ^{1}H NMR.[2]

Block Copolymerization (Figure 4D)

The ATRP cocktail (5 mL) with a target DP = 50 was prepared according to the general procedure by adding HOBiB solution (300 mM, 100 μL). The final concentrations were OEOMA$_{500}$ (300 mM), HOBiB (6.0 mM), EYH$_2$ (15 μM), CuBr$_2$ (0.3 mM), TPMA (0.9 mM), DMSO (10% v/v). Then 4.4 mL of the ATRP cocktail with a target DP = 50 (OEOMA$_{500}$) was added to a 1 dram (12/96 mm) vial equipped with a magnetic stirrer. The polymerization mixture was stirred at 500 rpm for 30 min under green LEDs (520 nm, 9.0 mW/cm^2). The macroinitiator p(OEOMA$_{500}$) was synthesized with 70% monomer conversion (M_n = 16 500, $Đ$ = 1.20). A sample (1 mL) was then taken from the post-polymerization mixture and used without further purification to prepare ATRP cocktail with OEOMA$_{300}$ monomer (DP$_T$ = 250) as follows. In a 5 mL volumetric flask, 450 mg of OEOMA$_{300}$ (average M_n = 300) was weighed. Crude p(OEOMA$_{500}$) solution (1 mL), CuBr$_2$ stock (200 μL), TPMA stock (100 μL), EYH$_2$ stock (50 μL) and 10X PBS solution (500 μL) were then added. Then 4.4 mL of the ATRP cocktail with a target DP = 250 (OEOMA$_{300}$) was added to a 1 dram (12/96 mm) vial equipped with a magnetic stirrer. The polymerization mixture was stirred at 500 rpm for 30 min under green LEDs (520 nm, 9.0 mW/cm^2). The sample was taken and analyzed by ^{1}H NMR and SEC techniques. SEC analysis showed a clear shift toward higher molecular weights without any shoulder or tailing at lower molecular weights (M_n = 51 000, $Đ$ = 1.18).

Low Volume Polymerizations (Table S1)

The ATRP cocktail (5 mL) with a target DP = 1000 was prepared according to the general procedure by adding HOBiB solution (15 mM, 100 μL). The final concentrations were OEOMA$_{500}$ (300 mM), HOBiB (0.3 mM), EYH$_2$ (15 μM), CuBr$_2$ (0.3 mM), TPMA (0.9 mM), DMSO

(10% v/v). Different volumes of ATRP cocktail (4400, 250, 150, or 50 μL) were then added to reaction vials of different volumes (see bottom photo). The polymerization mixtures ware irradiated for 30 min under green light LEDs (520 nm, 9.0 mW/cm^2). Samples were taken and analyzed by ^{1}H NMR and SEC techniques.

Table S1. Low volume ATRP[a]

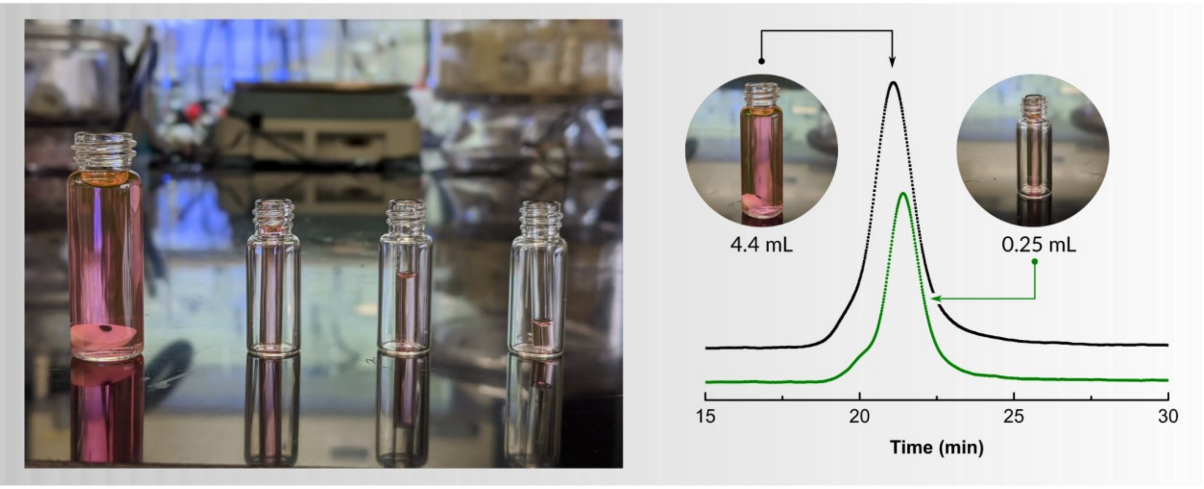

Entry	Volume (μL)	[b]S/Vol	[c]Rpm	[d]Conv. (%)	$M_{n,th}$ × 10^{-3}	[e]$M_{n,abs}$ × 10^{-3}	[f]$M_{n,app}$ × 10^{-3}	[f]$Đ$
1	4400	0.14	500	69	345	607	332	1.15
2	250	0.50	0	72	360	701	374	1.19
3	150	0.83	0	76	380	738	390	1.24
4	50	2.50	0	80	400	532	298	1.46

[a]Reaction conditions: [$OEOMA_{500}$]/[HOBIB]/[EYH_2/][$CuBr_2$]/[TPMA] = 1000/1/0.05/1.0/3.0, [$OEOMA_{500}$] = 300 mM, in 1X PBS with DMSO (10% v/v), irradiated for 30 min under green light LEDs (λmax = 520 nm, 9.0 mW/cm^2) in an open vial. [b]Surface area to volume ratio. [c]Stirring. [d]Monomer conversion was determined by using ^{1}H NMR spectroscopy. [e]Molecular weight ($M_{n,abs}$) was determined by Mark-Houwink calibration. [f]Apparent molecular weight ($M_{n,app}$) and dispersity ($Đ$) were determined by GPC analysis (DMF as eluent) calibrated to poly(methyl methacrylate) standards.

Synthesis of Protein Macroinitiator (BSA-iBB_{22})

ATRP initiators were covalently attached to the lysine groups of Bovine Serum Albumin (BSA) following the previously reported protocol.[3] BSA (1.0 g, 0.5 mmol of amine groups) was dissolved in 100 mM sodium phosphate buffer (pH = 8.0) at 0 °C. *N*-2-bromo-2-methylpropanoyl-*β*-alanine *N'*-oxysuccinimide ester (1.0 g, 3.0 mmol) dissolved in 1 mL of DMSO was then added dropwise. The mixture was stirred in a refrigerator for 4 h at 4 °C. The modified protein-macroinitiator was then dialyzed against 1X PBS for 48 hours at 4 °C using a 10 KDa molecular weight cut-off membrane, changing the dialysis buffer eight times. The dialyzed solution was lyophilized and stored at -20 °C. The number of lysines substituted with ATRP initiators (22/30) was determined by fluorescence spectroscopy with a fluorescamine assay, using a calibration curve of the native protein.

Supplementary Information

Synthesis of Protein-Polymer Biohybrid (Figure 4E)

Prior to polymerization, stock solutions of $CuBr_2$ (33.5 mg in 20.0 mL DMSO), TPMA (13.1 mg in 1.0 mL DMSO) and EYH_2 (9.7 mg in 10 mL DMSO) were prepared. The ATRP cocktail was then prepared as follows. In a 5 mL volumetric flask, 750 mg of $OEOMA_{500}$ was weighed. $CuBr_2$ stock (200 µL), TPMA stock (100 µL), EYH_2 stock (50 µL), DMSO (50 µL) and 10X PBS solution (500 µL) were then added. Finally, water was added to the mark on the volumetric flask, and the reaction mixture was stirred on a vortex. The reaction cocktail was transferred to another vial containing BSA-iBB_{22} (14.8 mg) and 3-morpholinopropane-1-sulfonic acid (10 mg). The protein macroinitiator (BSA-iBB_{22}) was dissolved completely. The final concentrations were $OEOMA_{500}$ (300 mM), BSA-iBB_{22} (34 µM), EYH_2 (15 µM), $CuBr_2$ (0.3 mM), TPMA (0.9 mM), DMSO (10% v/v). Then 4.4 mL of the ATRP cocktail was added to a 1 dram (12/96 mm) vial equipped with a magnetic stirrer. The polymerization mixture was stirred at 500 rpm for 30 min under green LEDs (520 nm, 9.0 mW/cm^2). The reaction vial was cooled by a stream of air flowing through the LED reactor. Sample was taken and analyzed by ^1H NMR. The protein-polymer bioconjugate was purified by dialysis using 30k DA molecular weight cut-off membrane for 48 h in 1X PBS. The dialyzed solution was lyophilized and analyzed by SEC-MALS using 1X DPBS as the eluent.

Synthesis of DNA-Polymer Biohybrid (Figure 4F)

Prior to polymerization, stock solutions of $OEOMA_{500}$ (0.5 M, 250 mg in water), $CuBr_2$ (56 mM, 14.4 mg in 1.0 mL of water/DMSO 1/1), TPMA (336 mM, 9.8 mg in 100 µL DMSO), EYH_2 (1.5 mM, 4.9 mg in 5 mL DMSO) were prepared. For the polymerization from model DNA initiator (T10-iBBr), $PEGMA_{500}$ stock (150 µL), $CuBr_2$ stock (1.33 µL), TPMA stock (0.67 µL), EYH_2 stock (2.5 µL), 10X PBS solution (25 µL), water (45.5 µL), and 25 µL of 5 mM DNA T10-iBBr (for DP_T = 600) was added in PCR tube and mixed thoroughly. The final concentrations were $OEOMA_{500}$ (300 mM), T10-iBBr (0.5 mM), EYH_2 (15 µM), $CuBr_2$ (0.3 mM), TPMA (0.9 mM), and DMSO (10% v/v). The ATRP cocktail was transferred into a glass insert in a 0.5 dram vial followed by irradiation of green light (520 nm, 3.7 mW/cm^2) for 30 min. The reaction vial was cooled by a stream of air flowing through the LED reactor. Sample was taken and analyzed by ^1H NMR. The DNA-polymer bioconjugate was purified by using 10K Amicon ultra centrifugal filter (*Millipore Sigma*), passing through *ChemMatrix* resin to remove eosin. The biohybrid was analyzed by SEC-MALS using 1X DPBS as an eluent.

Supplementary Information

ATRP in the Presence of Cells – Cytotoxicity Assay (Figure 4G)

Cytotoxicity and cell proliferation ware assessed using a direct CyQUANT® nucleic acid-sensitive fluorescence assay (*Thermo Fisher Scientific*) according to the manufacturer's instructions as described previously.[4] 100 μL aliquots of HEK293 cell suspension containing 1 × 10^6 cells/mL were plated in wells of a 96-well microplate (*Corning Inc.*,) and allowed to adhere overnight. Once the cells were attached, media was aspirated and cells were washed with 1X PBS (1X PBS solution was prepared from 10X PBS (*Thermo Fisher Scientific*) and DI water).

Prior to ATRP, stock solutions of $OEOMA_{500}$ (0.5 M, 250 mg in water), $CuBr_2$ (56 mM, 14.4 mg in 1.0 mL of water/DMSO 1/1), TPMA (336 mM, 9.8 mg in 100 μL DMSO), EYH_2 (1.5 mM, 4.9 mg in 5 mL DMSO), HOBiB (100 mM, 10.6 mg in 0.5 mL water) were prepared. In addition, stock solutions of CPADB (100 mM, 13.97 mg in 0.5 mL DMSO) and TEOA (100 mM, 14.9 mg in 1 mL water) were prepared for RAFT polymerization. For the photo-ATRP, final concentrations were $OEOMA_{500}$ (300 mM), HOBiB (1.5 mM), EYH_2 (15 μM), $CuBr_2$ (0.3 mM), TPMA (0.9 mM), DMSO (2% v/v) in 1X PBS. For the PET-RAFT, final concentrations were $OEOMA_{500}$ (300 mM), CPADB (1.5 mM), EYH_2 (15 μM), TEOA (0.9 mM), DMSO (2% v/v) in 1X PBS. Then 250 μL of the ATRP/RAFT cocktail was added to the cells in 96-well plate. The polymerization mixture was irradiated using the Lumidox 96-Well Green LED Array (520 nm, 20.0 mW/cm^2) for 10 min at room temperature. After polymerization, cells were washed two times with 1X PBS and stained with calcien AM dye (LIVE/DEAD™; Viability/Cytotoxicity Kit, *Invitrogen*) for 20 min at room temperature. Then, fluorescence from the calcien AM dye was measured with a TECAN spectrophotometer reader (*TECAN*). Cytotoxicity was assessed by normalizing fluorescence intensities to the non-treatment control group and plotted as percent viability.

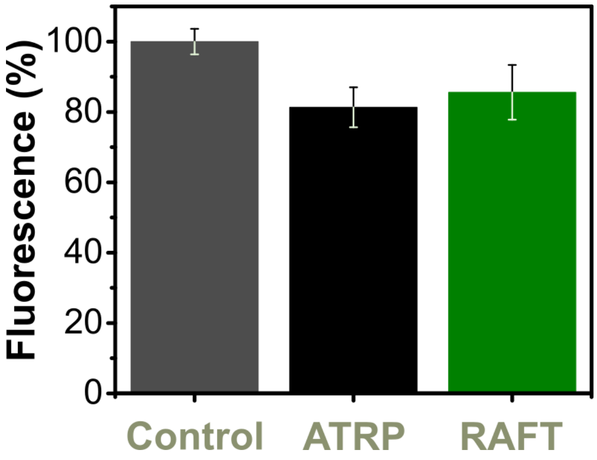

Figure S5. Biocompatibility of green-light-driven ATRP and PET-RAFT in the presence of HEK293 cells. (A) Cell viability was assessed using calcien AM staining. Cells were exposed to green light mediated polymerization (ATRP/RAFT) for 10 min at room temperature, washed and then assessed for viability. Bars indicate mean ± SEM (n=4), *$p \leq 0.05$ vs the control group.

General Procedure for EY/Cu-catalyzed ATRP of MA in DMSO

Prior to polymerization, stock solutions of MBiB (119.5 mg in 12 mL of MA), $CuBr_2$ (33.5 mg in 20.0 mL of DMSO), Me_6TREN (38 mg in 2.0 mL of DMSO) and EYH_2 (9.7 mg in 10 mL of DMSO) were prepared. The ATRP cocktail was then prepared as follows. MBiB stock solution in MA (2.5 mL), $CuBr_2$ stock solution (916 μL), Me_6TREN stock solution (500 μL), and EYH_2 stock solution (92 μL) were added to a 5 mL volumetric flask. Finally, DMSO was added to the mark on the volumetric flask, and the reaction mixture was stirred on a vortex. The final concentrations were MA (5.5 M), MBiB (27.5 mM), EYH_2 (27.5 μM), $CuBr_2$ (1.38 mM), Me_6TREN (8.25 mM). Then 4.4 mL of the ATRP cocktail was added to a 1 dram (12/96 mm) vial. The polymerization mixture was irradiated for 60 min under green light LEDs (520 nm, 9.0 mW/cm^2) without stirring. Samples were taken and analyzed by ^1H NMR and SEC techniques.

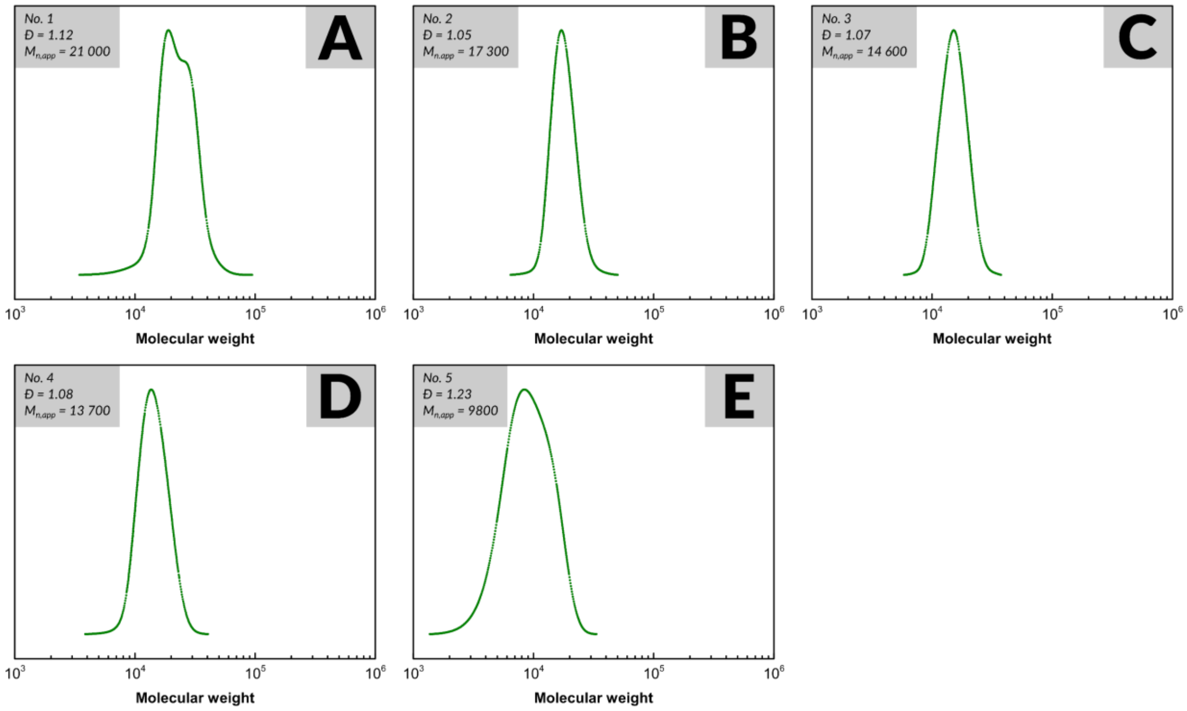

Figure S6. GPC traces for the polymerizations from Table 3.

Determination of Mark-Houwink Parameters of $OEOMA_{500}$ in DMF

PMMA and polystyrene are commonly used standards for the calibration of a GPC. For many polymers, no standards with sufficiently low dispersity and polymer range are readily available. Using a *Mark-Houwink* (MH) calibration, one can determine the molecular weight of a plethora of polymers using a PMMA or PS calibration curve. To obtain the corrected molecular weights, the MH parameters of the polymer under identical conditions are required. Often these values are reported, but it is possible to determine these values if it is possible to determine the molecular weight of the desired polymer through a secondary method. In the following paragraphs, we describe the process of determining the MH parameters of p($OEOMA_{500}$) in DMF with 0.05 M LiBr at 50 °C.

The hydrodynamic volumes of two polymers eluting at the same chromatographic retention time, are assumed to be identical According to the Flory-Fox equation, the hydrodynamic volume of a macromolecule is proportional to its intrinsic viscosity and molecular weight.[5]

$$\boldsymbol{V_h \propto \eta M} \qquad \textbf{Equation 1}$$

The empirical MH equation relates the intrinsic viscosity of a specific polymer to its molecular weight equation 2. Substituting the viscosity in equation 1 with the MH equation yields equation 3.[6]

$$\boldsymbol{\eta = KM^{a}} \qquad \textbf{Equation 2}$$

$$\boldsymbol{V_h \propto KM^{a}M = KM^{1+a}} \qquad \textbf{Equation 3}$$

The assumption that the hydrodynamic volumes of two polymers eluting at the same chromatographic retention time are assumed to be identical and thus leads to equation 4.

$$\boldsymbol{K_1 M_1^{1+a_1} = K_2 M_2^{1+a_2}} \qquad \textbf{Equation 4}$$

Rearranging equation 4 gives the molecular weight M_2 of the analyte.

$$\boldsymbol{\log M_2 = \frac{1}{1+a_2}\left(log\frac{K_1}{K_2}\right) + \frac{1+a_1}{1+a_2}\log M_1} \qquad \textbf{Equation 5}$$

Supplementary Information

Rewriting Equation (4) serves to establish a linear relationship between the logarithm of the molecular weight of one polymer (a standard) and that of another polymer, which after plotting gives a slope and intercept that can be converted to the MH parameters. The molecular weight of p(OEOMA$_{500}$) was determined by multi angle light scattering (MALS). The PMMA molecular weights and p(OEOMA$_{500}$) molecular weights used for the determination of the MH parameters are listed in Table S2 and plotted in Figure S5. The data is fit to equation 6. K_1 = 20.94 and a_1 0.642.

$$\boldsymbol{y = nx + m} \qquad \textbf{Equation 6}$$

with

$$\boldsymbol{n = \frac{1+a_1}{1+a_2}} \qquad \textbf{Equation}$$

and

$$\boldsymbol{m = \frac{1}{1+a_2}\left(log\frac{K_1}{K_2}\right)} \qquad \textbf{Equation 7}$$

Rewriting equation 6 and 7 to equations 8 and 9 gives the MH parameters K_2 = 354 and a_2 = 0.355.

$$\boldsymbol{a_2 = \frac{1+a_1}{n} - 1 = 0.355} \qquad \textbf{Equation 8}$$

$$\boldsymbol{K_2 = \frac{K_1}{10^{m(1+a_2)}} = 354} \qquad \textbf{Equation 9}$$

Using equation 5, these MH parameters give good estimates of the p(OEOMA$_{500}$) molecular weight (Table S2).

Table S2. Molecular weight of PMMA and p(OEOMA$_{500}$) with the same elution time.[a]

Entry	$M_{n,PMMA}$ (M_1)	Log(M_1)	$M_{n,MALS}$ p(OEOMA$_{500}$) (M_2)	Log(M_2)	$M_{n,abs}$ p(OEOMA$_{500}$)
1	18 000	4.26	15 000	4.18	17 800
2	29 800	4.47	32 300	4.51	32 700
3	30 400	4.48	32 300	4.51	33 500
4	30400	4.48	33 300	4.52	33 500
5	47 200	4.67	55 700	4.75	57 100
6	48 000	4.68	65 100	4.81	58 300
7	50 300	4.70	62 700	4.80	61 700
8	50 750	4.71	63 500	4.80	62 400
9	59 200	4.77	83 900	4.92	75 200
10	62 800	4.80	78 000	4.89	80 700
11	65 000	4.81	96 600	4.98	84 200
12	65 900	4.82	94 800	4.98	85 600
13	69 000	4.84	101 850	5.01	90 500
14	72 400	4.86	90 300	4.96	95 900
15	81 500	4.91	112 500	5.05	110 700
16	83 400	4.92	109 300	5.04	113 900
17	111 500	5.05	174 080	5.24	161 900
18	112 000	5.05	161 840	5.21	162 700
19	120 300	5.08	163 600	5.21	177 500
20	136 000	5.13	186 000	5.27	205 900
21	150 400	5.18	196 600	5.29	232 600
22	198 800	5.29	331 000	5.52	326 200

[a]Log used in the determination of the *Mark-Houwink* parameters of OEOMA$_{500}$ in DMF with 0.05 M LiBr, 50 °C.

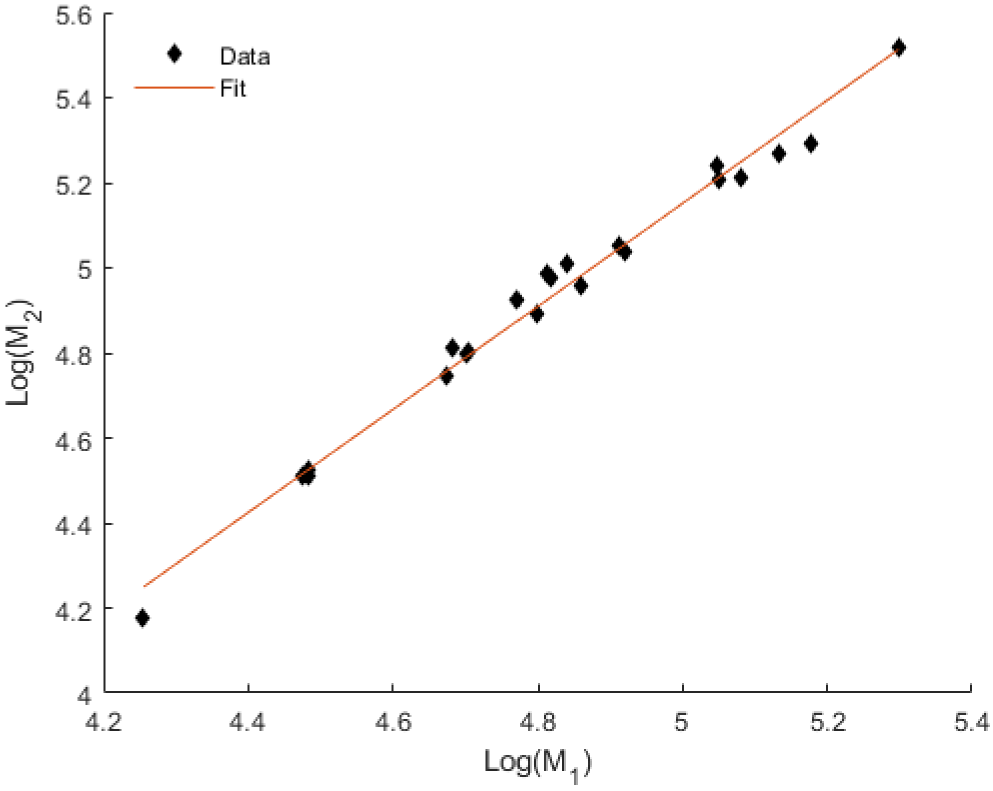

Figure S5. MH parameter fits to log(M2) vs. log(M1) data determined by SEC-MALS.

References

1. Averick, S. E.; Dey, S. K.; Grahacharya, D.; Matyjaszewski, K.; Das, S. R., Solid-Phase Incorporation of an ATRP Initiator for Polymer–DNA Biohybrids. *Angew. Chem. Int. Ed.* **2014,** *53* (10), 2739-2744.
2. Szczepaniak, G.; Piątkowski, J.; Nogaś, W.; Lorandi, F.; Yerneni, S. S.; Fantin, M.; Ruszczyńska, A.; Enciso, A. E.; Bulska, E.; Grela, K.; Matyjaszewski, K., An isocyanide ligand for the rapid quenching and efficient removal of copper residues after Cu/TEMPO-catalyzed aerobic alcohol oxidation and atom transfer radical polymerization. *Chem. Sci.* **2020,** *11* (16), 4251-4262.
3. Simakova, A.; Averick, S. E.; Konkolewicz, D.; Matyjaszewski, K., Aqueous ARGET ATRP. *Macromolecules* **2012,** *45* (16), 6371-6379.
4. Yerneni, S. S.; Lathwal, S.; Cuthbert, J.; Kapil, K.; Szczepaniak, G.; Jeong, J.; Das, S. R.; Campbell, P. G.; Matyjaszewski, K., Controlled Release of Exosomes Using Atom Transfer Radical Polymerization-Based Hydrogels. *Biomacromolecules* **2022,** *23* (4), 1713-1722.
5. Flory, P. J.; Fox, T. G., Treatment of Intrinsic Viscosities. *J. Am. Chem. Soc.* **1951,** *73* (5), 1904-1908.
6. Calibration. In *Modern Size-Exclusion Liquid Chromatography*, 2009; pp 193-229.
